# Multi-omics integration unravels four molecular subgroups of corticotroph pituitary neuroendocrine tumours with distinct clinicopathological features

**DOI:** 10.1038/s41467-026-76292-y

**Published:** 2026-08-04

**Authors:** Matthias Dottermusch, Alice Ryba, Antonia Gocke, Temor Rafiq, Celina Soltwedel, Tasja Lempertz, Linus Haberbosch, Simone Schmid, Luis Gustavo Perez-Rivas, Marily Theodoropoulou, Nesrin Uksul, Ulrich J. Knappe, Leonille Schweizer, Wolfgang Saeger, Jakob Matschke, Mateusz Bujko, Ulrich Schüller, Markus Glatzel, Jörg Flitsch, Franz L. Ricklefs, Julia E. Neumann

**Affiliations:** 1 Institute of Neuropathology, University Medical Center Hamburg-Eppendorf, Hamburg, Germany; 2 Center for Molecular Neurobiology (ZMNH), University Medical Center Hamburg-Eppendorf, Hamburg, Germany; 3 Mildred Scheel Cancer Career Center HaTriCS4, University Medical Center Hamburg-Eppendorf, Hamburg, Germany; 4 Department of Neurosurgery, University Medical Center Hamburg-Eppendorf, Hamburg, Germany; 5 Section of Mass Spectrometric Proteomics, University Medical Center Hamburg-Eppendorf, Hamburg, Germany; 6 Charité - Universitätsmedizin Berlin, corporate member of Freie Universität Berlin, Humboldt-Universität zu Berlin, and Berlin Institute of Health; Department of Endocrinology and Metabolism; European Reference Network on Rare Endocrine Diseases (ENDO-ERN), Berlin, Germany; 7 Department of Neuropathology, Charité University Medicine, Berlin, Germany; 8 Department of Medicine IV, LMU University Hospital, LMU Munich, Munich, Germany; 9 Department of Neurosurgery, Johannes Wesling Klinikum, Minden, Germany; 10 Edinger Institute (Institute of Neurology), University Hospital Frankfurt, Goethe University, Frankfurt am Main, Germany; 11 Institute of Pathology, University Medical Center Hamburg-Eppendorf, Hamburg, Germany; 12 Department of Molecular and Translational Oncology, Maria Sklodowska-Curie National Research Institute of Oncology, Warsaw, Poland; 13 Pediatric Hematology and Oncology, University Medical Center Hamburg-Eppendorf, Hamburg, Germany; 14 Children’s Cancer Research Center Hamburg, Hamburg, Germany

**Keywords:** Pituitary tumours, Proteomics, DNA methylation, Pituitary diseases, Classification and taxonomy

## Abstract

Corticotroph pituitary neuroendocrine tumours (PitNETs)/adenomas are heterogeneous sellar neoplasms. Currently established histopathological classification approaches are often considered limited in fully capturing the clinical and biological complexity of these tumours. Thus far, a molecular-based classification has not been established in corticotroph PitNETs. We compile molecular data of 270 corticotroph PitNETs (111 internal, 159 external), encompassing epigenome, transcriptome, and proteome profiles. Comprehensive integrative analyses are performed to identify, validate and characterise definitive molecular subgroups. Corticotroph PitNETs separate into four robust and clinicopathologically distinct molecular subgroups, which are broadly distinguishable by microscopy using SSTR1, GATA3 and SSTR5 immunohistochemistry. An integrated stratification model incorporating these molecular subgroups demonstrates significant prognostic utility. Our findings support the establishment of a refined molecular-based corticotroph PitNET classification, the full clinical value of which will require validation in prospective studies. To facilitate future research, we provide an easy-to-use epigenomic classifier for corticotroph PitNETs.

## Abstract (web summary)

Corticotroph pituitary neuroendocrine tumours (PitNETs)/adenomas are heterogeneous sellar neoplasms that lack molecular-based classification. By performing multi-omics analysis of 270 corticotroph PitNETs, the authors identify four distinct molecular subgroups.

## Introduction

Corticotroph pituitary neuroendocrine tumours (PitNETs), also known as corticotroph pituitary adenomas, are neoplasms of the anterior pituitary gland. Clinical manifestations range from clinically “silent” tumours to overt Cushing’s disease (CD) in functioning tumours. Upon detection, tumours may present as microscopic lesions or as large, invasive, and aggressive neoplasms. The postoperative disease course is variable with heterogeneity in tumour growth dynamics, recurrence rates and treatment responses^1–5^.

PitNET classification is currently based on pituitary lineage identity, determined through histomorphology and immunohistochemistry. The transcription factors TPIT, SF1, and PIT1 indicate cell lineage, with corticotroph PitNETs showing TPIT immunopositivity and SF1 and PIT1 immunonegativity. According to the current WHO classification, corticotroph PitNETs further divide into three histological subtypes. Sparsely granulated corticotroph tumours (SGCTs) are PAS-negative and exhibit variable ACTH expression. Densely granulated corticotroph tumours (DGCTs) are strongly PAS-positive and show intense ACTH immunoreactivity. Crooke cell tumours (CCTs) show tumour cells with Crooke’s hyaline change, characterised by ring-like accumulation of cytokeratins. The WHO classifications of 2021/2022 currently designate CCTs and “silent” corticotroph PitNETs as aggressive high-risk tumours^1,6^.

Current WHO classification standards for corticotroph PitNETs are often considered limited in reflecting clinically relevant tumour behaviour^7–10^. Furthermore, accurate histological subtyping of corticotroph PitNETs can be challenging, as the diagnostic criteria are primarily descriptive and some tumours can exhibit ambiguous or mixed granulation patterns^11,12^. Molecular analyses may allow to reclassify corticotroph PitNETs into potentially more meaningful and robust subgroups. The identification of recurrent somatic *USP8* mutations/variants represented a major advance in the molecular characterisation of corticotroph PitNET and was associated with a distinct clinicopathological profile marked by female predominance and functioning tumours^13–16^. Further investigations revealed distinct RNA and miRNA profiles in *USP8* variant corticotroph PitNETs^17,18^. With expanding case numbers and analyses of large-scale genome-wide epigenomic and transcriptomic data, three corticotroph PitNETs subgroups were described: (i) tumours harbouring *USP8* variants and exhibiting overt ACTH secretion; (ii) tumours with *USP8* reference sequences and greater invasiveness; and (iii) silent tumours with GATA3 expression^19,20^. While these previous studies provided valuable molecular insights, they were based on relatively confined tumour series and the potential clinical utility of a molecular classification has not yet been established in routine practice^1,8,19,20^.

In this study, we integrate internal with publicly available external cases, enabling an in-depth exploration of molecular subgroups in a total of 270 corticotroph PitNETs.

## Results

**Fig. 1:**
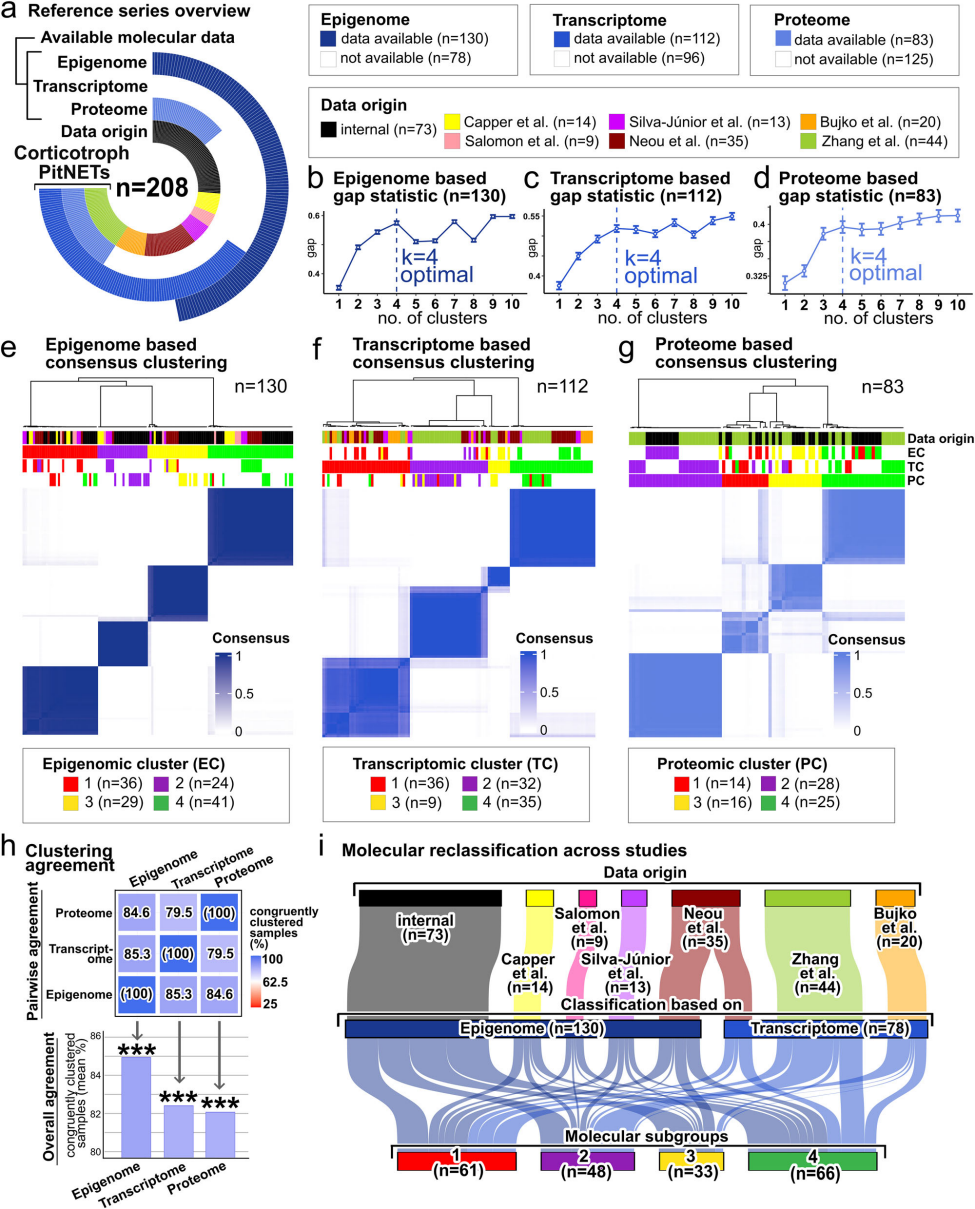
Molecular meta-analyses reveal four distinct subgroups of corticotroph PitNETs. **a** Sunburst chart shows an overview of all reference series samples, which were used for a comprehensive multi-omics meta-analysis. Available molecular data types and respective data origins are shown for each sample. **b-d** Gap statistics demonstrated the optimal number of clusters k to be 4 based independently on either epigenome (**b**), transcriptome (**c**) or proteome (**d**) data. Data points show the estimated gap statistic, error bars indicate ±1 Monte Carlo standard errors. **e-g** Consensus clustering based on epigenome (**e**), transcriptome (**f**) and proteome (**g**) data showed the distribution of samples among the four molecular clusters. Batch effects relating to different study origins were not apparent. **h** Clustering agreement was based on the percentage of samples (*n* = 117) with coherent clustering results using the three different molecular data types. Clustering agreement was significantly higher than would be expected by chance and epigenome-based clustering showed the highest overall agreement with the other two data types. *p*-values were calculated using Chi-square test and adjusted for multiple comparisons using Bonferroni correction: ***p < 0.001. Epigenome: *p* = 8.65533e-32, *n* = 73; Transcriptome: *p* = 8.09079e-31, *n* = 78; Proteome: *p* = 1.40039e-32, *n* = 83. **i** Considering the results shown in (**b**-**h**), the 208 corticotroph PitNETs were reclassified into the four molecular subgroups 1-4 based on DNA methylation data, or -when unavailable- RNA expression data. Sample sizes (n) indicate the number of tumour samples. All analyses shown were based on reference series samples for which corresponding data were available. Source data are provided as a Source Data file.

We compiled a reference series of 208 molecularly characterised corticotroph PitNETs consisting of 73 internal cases and 135 samples derived from public deposits [Bujko et al.^17^ (*n* = 20); Neou et al.^19^ (*n* = 35); Capper et al.^21^ (*n* = 14); Salomon et al.^22^ (*n* = 9); Zhang et al.^23^ (*n* = 44); Silva-Júnior et al.^24^ (*n* = 13)]. In total, DNA methylation data was available in 130 tumours, RNA expression data was available in 112 tumours and protein data was available in 83 tumours (Fig. 1a and Supplementary Data 1−2).

### Corticotroph PitNETs split into four distinct molecular subgroups

Molecular data of tumour samples were integrated across studies. We used gap statistic to determine the optimal number of sample clusters separately for epigenomic, transcriptomic, and proteomic data. In all three datasets, an optimal number of *k* = 4 clusters was determined (Fig. 1b–d and Supplementary Data 3a–c). Consensus clustering revealed segregation of samples without apparent batch effects relating to sample origins (Fig. 1e–g). Unsupervised dimension reduction via UMAP plotting confirmed samples grouping into distinct molecular clusters (Supplementary Fig. 1a–c). Clustering agreement among the three datasets was congruent and most robust using epigenome data, followed by transcriptome and proteome data (Fig. 1h). Therefore, we proceeded to reclassify all 208 samples based on DNA methylation, or -when unavailable-, RNA expression data- into four subgroups 1 (*n* = 61), 2 (*n* = 48), 3 (*n* = 33) and 4 (*n* = 66) (Fig. 1i).

### Characterisation of genomic and epigenomic features

**Fig. 2:**
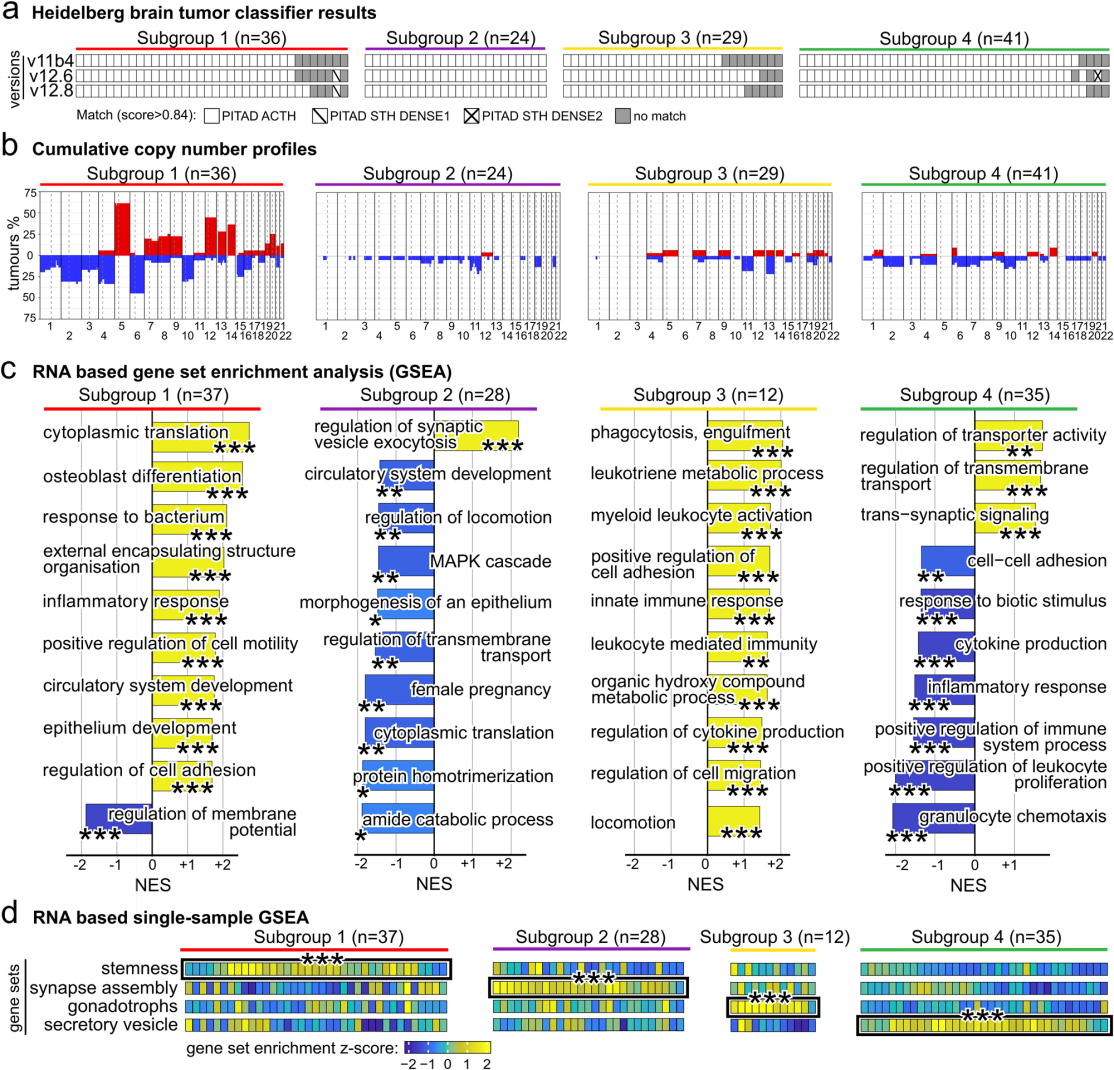
Epigenomic, genomic and transcriptomic features of corticotroph molecular subgroups. **a** The Heidelberg brain tumour classifier (v11b4, v12.6, and v12.8) confirmed corticotroph PitNET diagnosis based on DNA methylation signatures in most cases across the four molecular subgroups 1–4. **b** Cumulative copy number profiles of the molecular subgroups 1-4 are shown. Subgroup 1 evidently harboured the most frequent and abundant copy number alterations. **c** RNA based gene set enrichment analyses (GSEA) across the four subgroups revealed specific gene expression profiles. Gene ranking was based on median fold changes of one subgroup compared to the other three. The top 10 most significantly enriched gene sets of each subgroup are shown. Permutation-based gene set enrichment analysis. Gene ontology terms were reduced to remove semantic redundancy. Abbreviation: NES = normalised enrichment score. **d** RNA based single-sample GSEA revealed significant enrichment of the gene sets “stemness” (*p* = 2.783359e-04), “synapse assembly” (*p* = 1.276375e-09), “gonadotrophs” (*p* = 4.699951e-04), and “secretory vesicle” (*p* = 1.288816e-11), in subgroups 1-4, respectively. Two-sided Student’s *t*-test. Sample sizes (n) indicate the number of tumour samples. In (**c**) and (**d**), *p*-values were adjusted for multiple comparisons using Bonferroni correction: not significant (ns) *p* ≥ 0.05, *p < 0.05, **p < 0.01, ***p < 0.001. All analyses shown were based on reference series samples for which corresponding data were available. Source data are provided as a Source Data file.

The previously published Heidelberg brain tumour classifier (versions 11b4, 12.6 and 12.8) was used to calculate methylation class (mc) predictions in all samples with available epigenomic data^21,25^. The classifier computed scores >0.84 for the mc “PITAD ACTH” -which corresponds to corticotroph PitNETs- in 86% of subgroup 1 tumours, in 100% of subgroup 2 tumours, in 90% of subgroup 3 tumours and in 93% of subgroup 4 tumours (*n* = 31/36, 24/24, 26/29, and 38/41, respectively). All remaining tumours were classified as “no matches” in at least one classifier version (Fig. 2a). In summary, the classifier confirmed corticotroph PitNET diagnosis in the large majority samples, yet did not allow for a distinction of the subgroups 1–4. Cumulative copy number profiles (CCNPs) revealed highly abundant chromosomal alterations in subgroup 1 tumours, with frequent gains in chromosomes 5, 7-9, 12, 13q, 14q and 20, and frequent losses in chromosomes 2, 4, 6, 10 and 15q. Subgroup 2–4 tumours showed rather quiet CCNPs with infrequent alterations (Fig. 2b).

### Characterisation of transcriptome and proteome profiles

RNA-based gene set enrichment analyses (GSEA) were performed to identify subgroup-specific transcriptome signatures (Fig. 2c, Supplementary Data 4a–d). Significantly positively enriched terms in subgroup 1 mainly related to regulation of cellular differentiation, development, and growth (normalised enrichment scores (NES) = 1.39-2.6, *p* < 0.001). In subgroup 2, the term “regulation of synaptic vesicle exocytosis” was significantly positively enriched (NES = 2.2, *p* < 0.001). In subgroup 3, positive GO enrichment was mainly related to the regulation of immune response and cell migration (NES = 1.45-2.09, *p* < 0.001). In subgroup 4, terms associated with transmembrane signal transduction were among the significantly positively enriched results (NES = 1.52-1.83, *p* < 0.001). To further solidify the molecular signatures of the subgroups 1–4, we performed RNA-based single-sample GSEA, which demonstrated robust and highly significant enrichment of the gene sets “stemness”, “synapse assembly”, “gonadotrophs” and “secretory vesicle”, respectively (*p* < 0.001; Fig. 2d and Supplementary Data 5a–d). Interrogation of proteome data supported our transcriptome-based findings, and provided additional insights, such as a high abundance of mitochondrial proteins in subgroup 4 compared to the others (Supplementary Fig. 2, Supplementary Data 6a–d).

### Characterisation of histomorphology

**Fig. 3:**
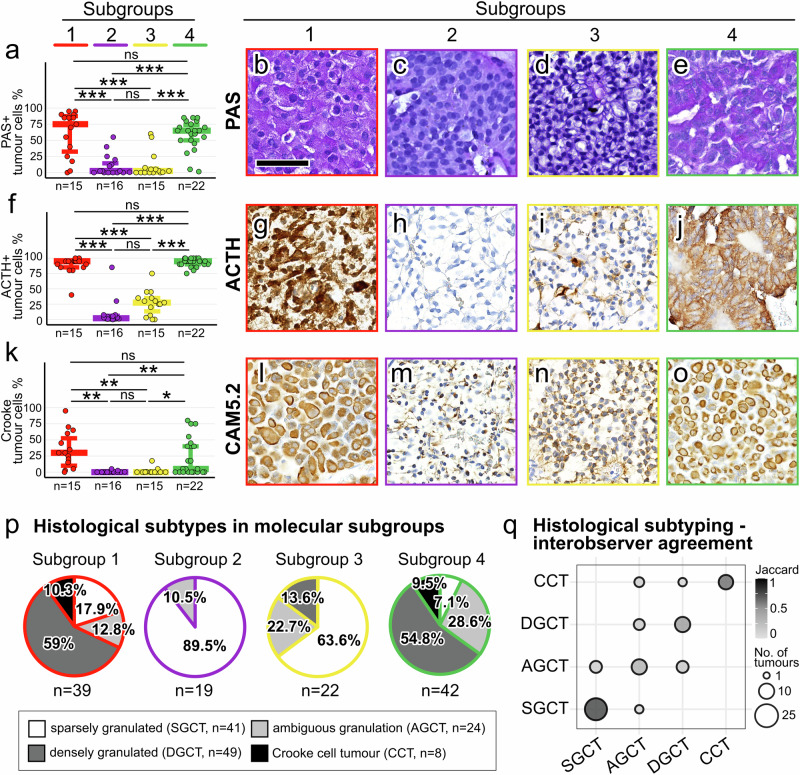
Association of WHO-defined histological subtypes and molecular subgroups is limited in corticotroph PitNETs. **a–e** PAS positivity was mainly found in tumours of subgroups 1 and 4. Representative histopathological images of subgroup 1 (*n* = 15), 2 (*n* = 16), 3 (*n* = 15), and 4 (*n* = 22) tumours are shown in (**b–e**), respectively. **f–j** ACTH immunopositivity was mainly found in subgroups 1 and 4. Representative histopathological images of subgroup 1 (*n* = 15), 2 (*n* = 16), 3 (*n* = 15), and 4 (*n* = 22) tumours are shown in (**g–j**), respectively. **k–o** Tumour cells with circular CAM5.2 staining corresponding to Crooke cells were mainly encountered in subgroups 1 and 4. Representative histopathological images of Crooke cell tumours of subgroup 1 (*n* = 4) and subgroup 4 (*n* = 4) are shown in l and o, respectively. Representative histopathological images of subgroup 2 (*n* = 16) and subgroup 3 (*n* = 15) tumours are shown in (**m**) and (**n**), respectively. **p** Pie graphs show the proportions of histological subtypes among the four molecular subgroups across studies. Densely granulated corticotroph PitNETs (DGCTs) and Crooke cell tumours (CCTs) were mainly encountered in subgroups 1 and 4. Sparsely granulated corticotroph PitNETs (SGCTs) were mainly encountered in subgroups 2 and 3. Corticotroph PitNETs with ambiguous granulation patterns (AGCT), were encountered in all four molecular subgroups. Histological subtyping of all internal cases was based on the data shown in (**a**), (**f**), and (**k**). Histological subtypes of all external cases were assigned as reported in their original publications. **q** Agreement in histological subtyping of two independent observers is shown for all internal cases. Cohen’s kappa = 0.577, *p* = 6.66e-16, *n* = 68. Scale bar is 50 µm in all histological images. Sample sizes (n) indicate the number of tumour samples. In a, f, and k, horizontal bars indicate the interquartile range (IQR, 25th-75th percentiles), and central lines mark medians. Percentages based on the mean quantifications of two independent observers are shown. *p*-values were calculated using two-sided Student’s *t-*test and adjusted for multiple comparisons using Bonferroni correction: not significant (ns), *p* ≥ 0.05, *p < 0.05, **p < 0.01, ***p < 0.001. All analyses shown were based on reference series samples for which corresponding data were available. Source data are provided as a Source Data file.

Current diagnostic standards of the WHO require histological subtyping of corticotroph PitNETs as SGCTs, DGCTs, or CCTs, using PAS-staining and immunohistochemistry for ACTH and CAM5.2. PAS and ACTH staining, as well as the presence of Crooke tumour cells were semi-quantitatively assessed in all internal cases with sufficiently available tissue (*n* = 68), based on the mean scorings of two independent observers. PAS positivity, ACTH expression and Crooke tumour cells were most prevalent in tumours of subgroups 1 and 4 (*p* < 0.05 compared to subgroups 2 and 3). No significant differences were detected when comparing subgroup 1 with 4 (*p* = 1), or when comparing subgroup 2 with 3 (*p* > 0.16; Fig. 3a–o).

Based on histological features of internal cases and the annotations of external samples, we investigated the proportions of the different histological subtypes within the molecular subgroups (Fig. 3p). Subgroups 1 and 4 harboured mostly DGCTs (59%, 23/39 and 54.8%, 23/42, respectively). Subgroups 2 and 3 harboured mostly SGCTs (89.5%, 17/19 and 63.6%, 14/22, respectively). Tumours with ambiguous granulation were found in all four subgroups (12.8%, 5/39; 10.5%, 2/19; 22.7%, 5/22 and 28.6%, 12/42 in subgroups 1-4, respectively). CCTs were found in subgroups 1 and 4 (10.3%, 4/39 and 9.5%, 4/42, respectively). Of note, comparing the staining assessments of individual observers demonstrated limited agreement in histopathological subtyping with high rates of tumours showing ambiguous granulation (Cohen’s kappa = 0.577, *p* = 6.66e-16; Fig. 3q and Supplementary Data 7).

### SSTR1, GATA3 and SSTR5 expression associates with corticotroph molecular subgroups

**Fig. 4:**
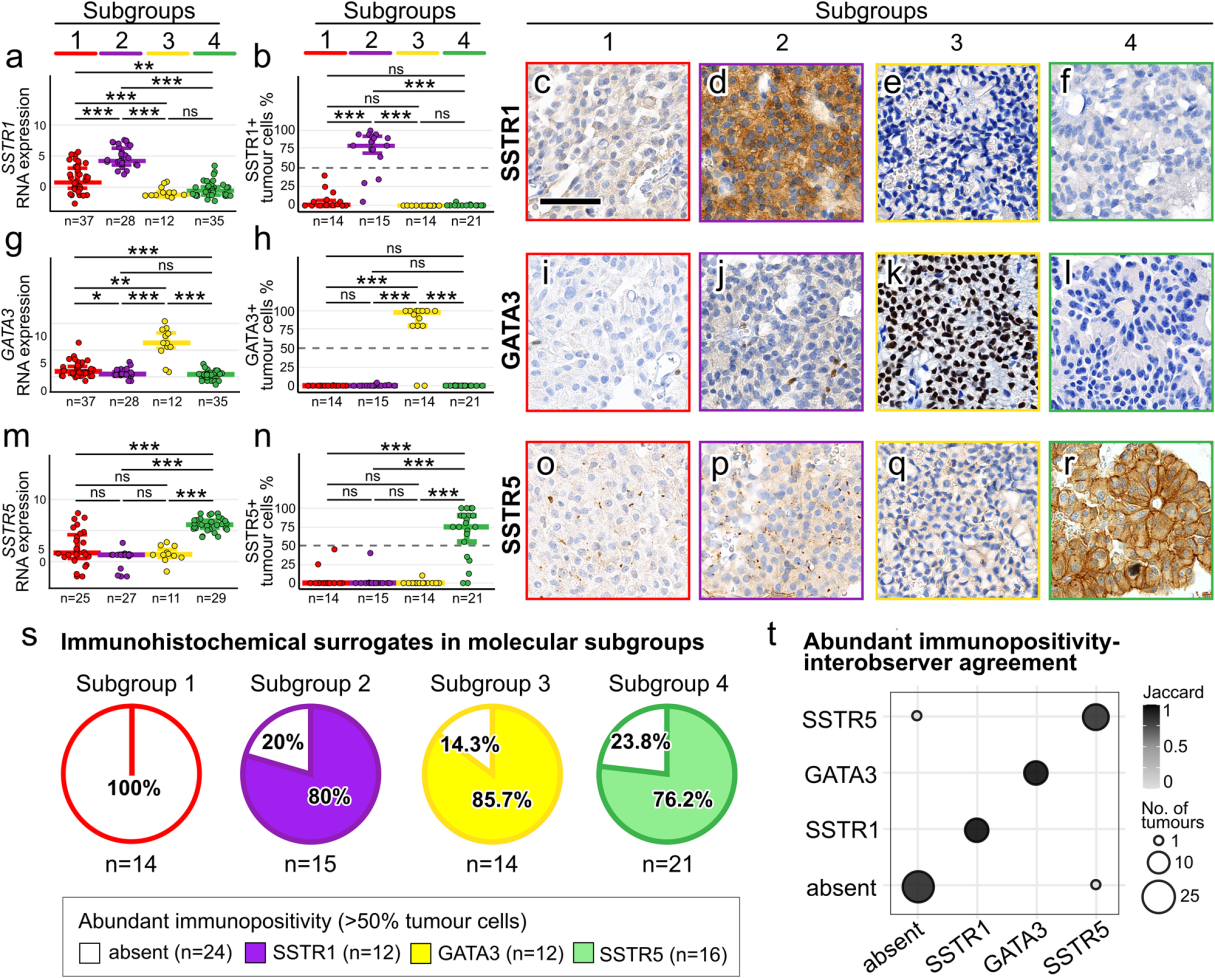
Immunohistochemical surrogate markers distinguish corticotroph molecular subgroups. **a** RNA expression levels of *SSTR1* were significantly higher in subgroup 2. **b-f** SSTR1 immunopositivity was mostly encountered in subgroup 2 tumours. A representative histopathological image of a subgroup 2 tumour with abundant SSTR1 immunopositivity (*n* = 12) is shown in (**d**). Cytoplasmic immunoreactivity was considered positive. Representative histopathological images of subgroup 1 (*n* = 14), 3 (*n* = 14), and 4 (*n* = 21) tumours are shown in (**c**,**e**,**f**), respectively. **g** RNA expression levels of *GATA3* were significantly higher in subgroup 3. **h–l** GATA3 immunopositivity was mostly encountered in subgroup 3 tumours. A representative histopathological image of a subgroup 3 tumour with abundant GATA3 immunopositivity (*n* = 12) is shown in (**k**). Nuclear immunoreactivity was considered positive. Representative histopathological images of subgroup 1 (*n* = 14), 2 (*n* = 15), and 4 (*n* = 21) tumours are shown in (**i**,**j**,**l**), respectively. Note the occasional presence of vessel-associated GATA3-positive cells in (**i**,**j**) which were not recognised as relevant immunopositivity. **m** RNA expression levels of *SSTR5* were significantly higher in subgroup 4. Of note, *SSTR5* transcripts were detected only in a subset of cases. **n–r** SSTR5 immunopositivity was mostly encountered in subgroup 4 tumours. A representative histopathological image of a subgroup 4 tumour with abundant SSTR5 immunopositivity (*n* = 16) is shown in (**r**). Membranous immunoreactivity was considered positive. Representative histopathological images of subgroup 1 (*n* = 14), 2 (*n* = 15), and 3 (*n* = 14) tumours are shown in (**o**,**p**,**q**), respectively. Note the scant granular staining pattern for SSTR5 in (**o**,**p**), which was not recognised as relevant immunopositivity. **s** Mutually exclusive abundant immunostaining for either SSTR1, GATA3 or SSTR5 was highly specific for tumours of the subgroups 2, 3 and 4, respectively. Subgroup 1 tumours did not show abundant immunostaining for any of these markers. Data shown in (**b**,**h**,**n**) provided the basis for the shown categorisations. **t** Agreement in assessing abundant immunopositivity for SSTR1, GATA3 and SSTR5 of two independent observers is shown for all internal cases. Cohen’s kappa = 0.961, *p* = 1.14e-47, *n* = 64. Scale bar is 50 µm in all histological images. Sample sizes (n) indicate the number of tumour samples. In (**a**,**b**,**g**,**h**,**m**,**n**), horizontal bars indicate the interquartile range (IQR, 25–75th percentiles), and central lines mark medians. In (**b**,**h**,**n**), percentages based on the mean quantifications of two independent observers are shown. *p*-values were calculated using two-sided Student’s *t*-test and adjusted for multiple comparisons using Bonferroni correction: not significant (ns) *p* ≥ 0.05, *p < 0.05, **p < 0.01, ***p < 0.001. All analyses shown were based on reference series samples for which corresponding data were available. Source data are provided as a Source Data file.

We investigated the transcriptome data for potential surrogate markers for the molecular subgroups based on differentially expressed genes (Supplementary Data 3b) and previously published reports^19,20,26,27^. Immunopositivity was again assessed based on the mean scorings of two independent observers for all internal tumours with sufficiently available tissues (*n* = 64). *SSTR1* RNA levels were highest in subgroup 2 tumours (*p* < 0.001 vs. all other subgroups and Fig. 4a) and we found enhanced SSTR1 immunopositivity in this subgroup (*p* < 0.001 vs. all other subgroups, Fig. 4b–f). *GATA3* RNA levels were highest in subgroup 3 tumours (*p* < 0.01 vs. all other subgroups; Fig. 4g) and nuclear GATA3 immunopositivity was observed almost exclusively in this subgroup (*p* < 0.001 vs. all other subgroups, Fig. 4h–l). Finally, *SSTR5* RNA levels were highest in subgroup 4 tumours (*p* < 0.001 vs. all other subgroups; Fig. 4m) and we found enhanced SSTR5 immunopositivity in this subgroup (*p* < 0.001 vs. all other subgroups; Fig. 4n–r).

Considering abundant immunopositivity only (defined as >50% positive tumour cells), 100% (14/14) of subgroup 1 tumours were void of abundant SSTR1, GATA3 and SSTR5 staining. In subgroup 2, 80% (12/15) of tumours showed abundant SSTR1 expression. In subgroup 3, 85.7% (12/14) of tumours showed abundant GATA3 expression. In subgroup 4, 76.2% (16/21) of tumours showed abundant SSTR5 expression. Abundant immunopositivity for either SSTR1, GATA3 or SSTR5 was therefore mutually exclusive and specific for the subgroups 2-4, respectively (Fig. 4s). Of note, interobserver agreement in assessing the presence of abundant immunopositivity for SSTR1, GATA3 or SSTR5 was notably higher compared to histological subtyping (Cohen’s kappa = 0.961, *p* = 1.14e-47, Fig. 4t, Supplementary Data 7).

### Validation of four corticotroph molecular subgroups

Next, we compiled a validation series of 62 corticotroph PitNETs, with global DNA methylation data for every sample (Supplementary Fig. 3a and Supplementary Data 1,2). The series comprised 38 additional internally processed samples as well as 24 publicly available samples derived from Belakhoua et al.^28^. Gap statistics again showed *k* = 4 as the optimal number of epigenomic clusters in the validation series, and the Heidelberg classifier confirmed corticotroph lineages across the four subgroups (Supplementary Fig. 3b–d). Additionally, we were able to confirm abundant genomic alterations in subgroup 1 tumours (Supplementary Fig. 3e) and that, in contrast to granulation patterns, immunopositivity for SSTR1, GATA3 and SSTR5 was highly specific for tumours of subgroups 2, 3, and 4, respectively. Again, interobserver agreement was higher for assessing abundant surrogate marker immunopositivity than it was for histological subtyping (Supplementary Fig. 3f–i and Supplementary Data 7). Of note, epigenome-based clustering via UMAP plots, merging the samples of the validation (n = 62) with those of the reference series (*n* = 130), robustly reproduced the four molecular subgroups and confirmed stable epigenome profiles regardless of prior therapeutic interventions (Supplementary Fig. 4).

### Corticotroph molecular subgroups relate to distinct patient and tumour features

**Fig. 5:**
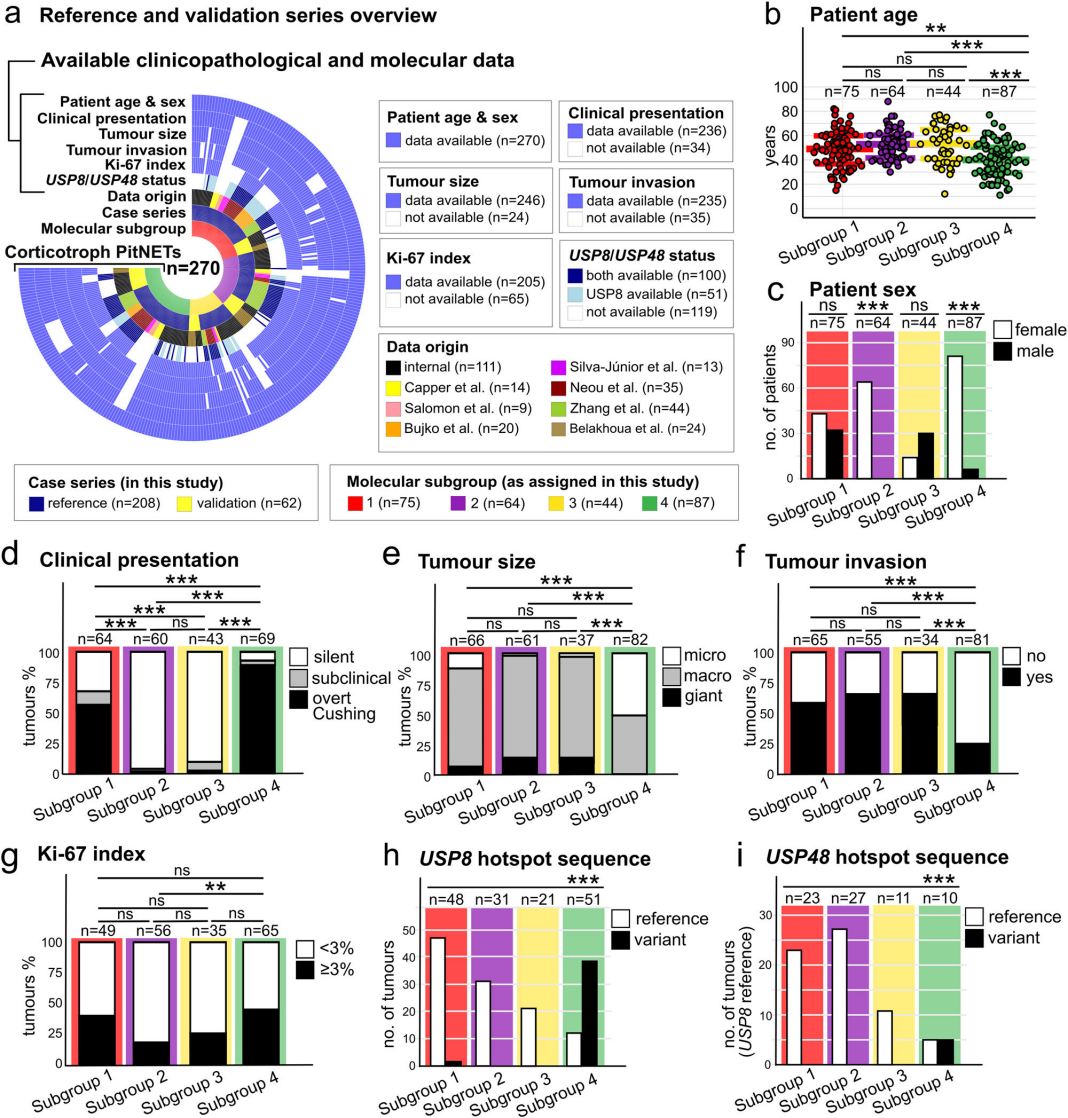
Patient and tumour features of corticotroph molecular subgroups. **a** Sunburst chart shows an overview of all samples and available data of the reference and validation series, which were used to investigate clinicopathological and molecular features across the molecular subgroups. **b** Patients with subgroup 4 tumours were significantly younger. Horizontal bars indicate the interquartile range (IQR, 25–75th percentiles), with central lines marking medians. Two-sided Student’s *t-*test. **c** Females were significantly overrepresented in subgroups 2 and 4. Chi-square test (expected ratio 1.0). **d** The four molecular subgroups showed differing clinical presentations in terms of functional status. Silent: No clinical or biochemical evidence of hormone secretion; Subclinical: Biochemical, but no clinical evidence of Cushing’s disease (CD); Overt Cushing: Clinically manifest CD. Two-sided Wilcoxon rank-sum test. **e** Tumours were significantly smaller in subgroup 4. PitNETs were classified by maximum diameter. Micro: Microtumours/-adenomas (< 1 cm); Macro: Macrotumours/-adenomas (1–4 cm); Giant: Giant tumours/adenomas (> 4 cm). Two-sided Wilcoxon rank-sum test. **f** Subgroup 4 tumours showed significantly less invasive growth. Two-sided Wilcoxon rank-sum test. **g** Increased proliferative activity, defined as ≥3% Ki-67-index, was most frequently encountered in subgroup 4 tumours, followed by tumours of subgroups 1,3, and 2. Two-sided Wilcoxon rank-sum test. **h** *USP8* hotspot variants were almost exclusively found in subgroup 4 tumours. Two-sided Fisher’s exact test. **i** *USP48* hotspot variants were exclusively found in subgroup 4 tumours. The shown analysis included only tumours with known *USP8* hotspot reference sequence. Two-sided Fisher’s exact test. In all panels, sample sizes (n) indicate the number of patients/tumour samples. *p*-values were adjusted for multiple comparisons using Bonferroni correction: not significant (ns) *p* ≥ 0.05, *p < 0.05, **p < 0.01, ***p < 0.001. All analyses shown were based on reference and validation series samples, for which corresponding data were available. Source data are provided as a Source Data file.

**Fig. 6:**
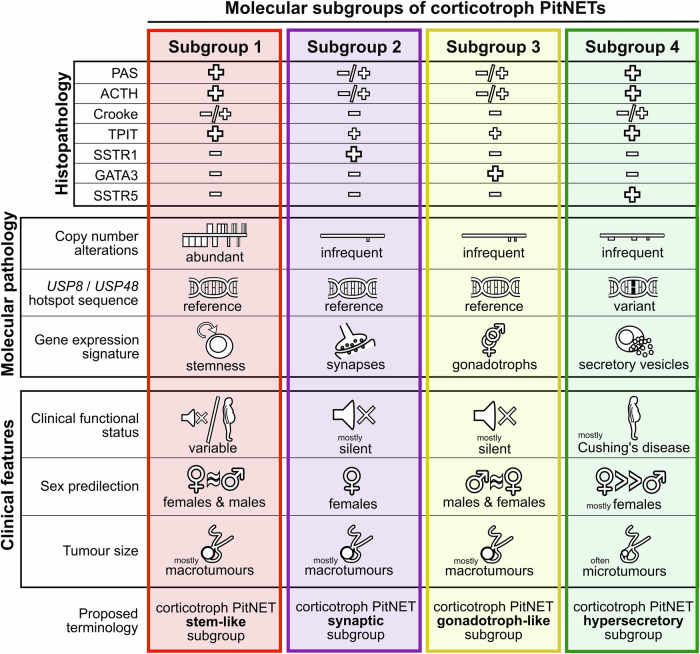
Main features of the corticotroph molecular subgroups. Simplified overview of clinicopathological and molecular features of the corticotroph PitNET molecular subgroups.

To further characterise clinicopathological and molecular features of the four molecular subgroups, we merged all data of the reference and the validation series (Fig. 5a and Supplementary Data 1−2). Patients with subgroup 4 tumours were significantly younger than those with tumours of other subgroups (median age: 49, 53, 53.5, and 40 years in subgroups 1-4, respectively, *p* < 0.01; Fig. 5b). There was a significant overrepresentation of female patients in subgroup 2 (64/64, 100%, *p* < 0.001) and subgroup 4 (81/87, 93.1%, *p* < 0.001, Fig. 5c). In terms of clinical presentation, subgroup 1 included 56.2% patients with overt CD (36/64), 10.9% with subclinical CD (7/64), and 32.8% with silent tumours (21/64). Subgroups 2 and 3 mostly included silent tumours (96.7% (58/60) and 90.7% (39/43), respectively) and subgroup 4 manifested mostly with overt CD (89.9%, 62/69) (subgroup 2 vs 3: *p* = 1; all other comparisons: *p* < 0.001; Fig. 5d). Subgroup 4 included the most microtumours (51.2% in subgroup 4, compared to 12.1%, 1.6%, and 2.7% in subgroups 1–3, respectively; 42/82, 8/66, 1/61, 1/37, *p* < 0.001; Fig. 5e). Tumour invasion was detected in 58.5% of subgroup 1 (38/65), 67.3% of subgroup 2 (37/55), and 67.6% of subgroup 3 (23/34), but only in 23.5% of subgroup 4 tumours (19/81; *p* < 0.001 subgroup 4 vs 1,2 and 3; Fig. 5f). Proliferative activity determined via Ki-67 immunostaining showed that high Ki-67-indices ≥3% were detected in 40.8% of subgroup 1 (20/49), 17.9% of subgroup 2 (10/56), 25.7% of subgroup 3 (9/35) and 47.7% of subgroup 4 tumours (31/65, *p* = 1, *p* = 0.003 and *p* = 0.2 comparing subgroup 4 with 1–3, respectively; Fig. 5g). We also investigated nuclear expression of the pituitary transcription factors TPIT, SF1 and PIT1. TPIT staining was most abundant in subgroups 1 and 4 (*p* < 0.01 compared to subgroups 2 and 3, Supplementary Fig. 5a–e). Variable SF1 expression was detected in 22.3% of cases (21/94) with similar distribution among the molecular subgroups (*p* > 0.14 and Supplementary Fig. 5f–j). None of the tumours showed nuclear PIT1 expression (Supplementary Fig. 5k–o). *USP8* hotspot variants were almost exclusively found in subgroup 4 [74.5%, 38/51, *p* < 0.001; Fig. 5h). Further investigating all tumours with confirmed absence of *USP8* variants revealed that *USP48* hotspot variants were exclusively found in subgroup 4 [50%, 5/10; *p* < 0.001; Fig. 5i). In summary, the four molecular subgroups showed distinct clinicopathological and molecular features (Fig. 6).

### Incorporation of corticotroph molecular subgroups into an integrated prognostic stratification model

To examine the potential prognostic value of the molecular subgroups, clinical trajectories were inferred from therapeutic interventions, reported tumour behaviour and at least 6 months of follow-up data. Cases were grouped as showing “initial remission” (no second intervention after initial tumour resection, no biochemical or imaging evidence of residual disease), “repeated surgeries / persistent or recurrent disease” (more than one tumour resection and/or evidence of disease at last follow-up), or “multimodal therapy / aggressive disease” (additional interventions after tumour resection in the form of radiotherapy and/or chemotherapy, and/or tumour aggressiveness according to original publications).

**Fig. 7:**
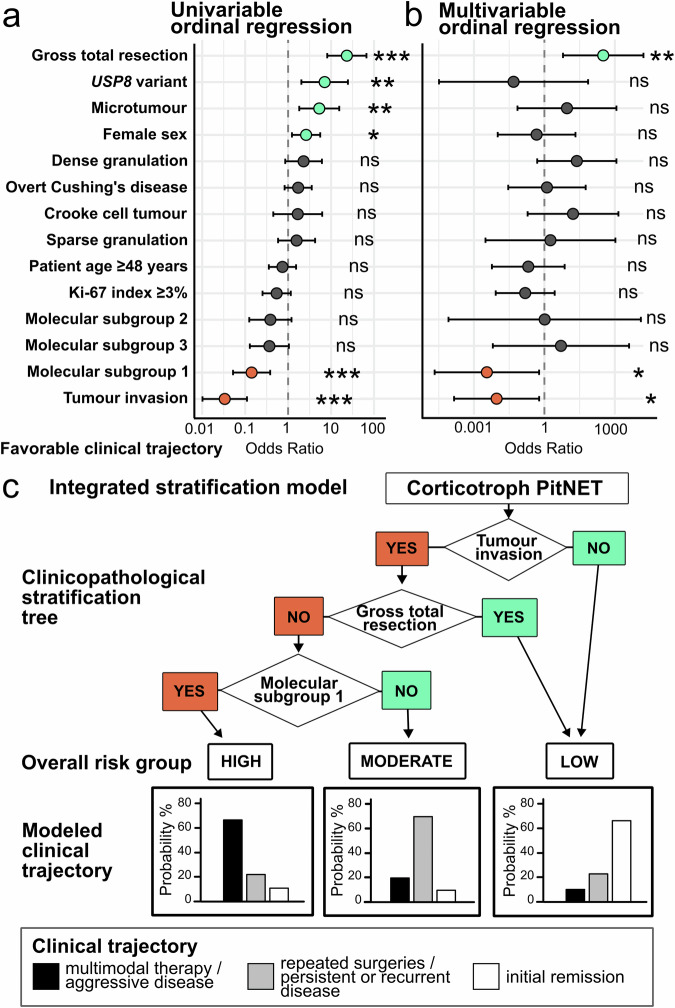
An integrated stratification model incorporates molecular subgroups for prognostication. **a**,**b** Results of univariable (**a**) and multivariable (**b**) ordinal regression analysis based on 106 samples of the reference (*n* = 80) and validation series (*n* = 26), for which sufficient follow-up data were available. Outcome variables were defined as ordinally ranked clinical trajectories. Categories not shown served as the reference categories for their respective variables. Age was dichotomised at 48 years, corresponding to the median patient age in the analysed cases. Odds ratios (ORs) with 95% confidence intervals (CIs) are shown. Not significant (ns) *p* ≥ 0.05, *p < 0.05, **p < 0.01, ***p < 0.001. **c** A decision tree displaying an enhanced stratification scheme for predicting clinical trajectories was constructed using a recursive partitioning algorithm. The decision tree considered tumour invasion, gross total resection and molecular subgroup 1 in hierarchical order, culminating into three overall risk groups labelled “high”, “moderate”, and “low”. Source data are provided as a Source Data file.

We performed ordinal regression to assess associations of clinicopathological and molecular features with clinical trajectories. In the univariable model, tumour invasion (OR = 0.033, 95% CI: 0.01–0.11, *p* < 0.001) and molecular subgroup 1 (OR = 0.14, 95% CI: 0.05–0.38, *p* < 0.001) were identified as significant negative prognostic predictors. In contrast, female sex (OR = 2.62, 95% CI: 1.23–5.58, *p* = 0.013), microtumours (OR = 5.3, 95% CI: 1.82–15.48, *p* = 0.002), *USP8* variant (OR = 7.09, 95% CI: 2.03–24.68, *p* = 0.002), and gross total resection (GTR, OR = 23.29, 95% CI: 8.13–66.69, *p* < 0.001) were associated with a more favourable clinical trajectory. No significant associations were observed for the remaining variables Ki-67, age, histological subtype or clinical presentation (all *p* > 0.05; Fig. 7a).

In multivariable ordinal regression including all aforementioned variables, only tumour invasion (adjusted OR = 0.01, 95% CI: 0.0001–0.6, *p* = 0.028), molecular subgroup 1 (adjusted OR = 0.004, 95% CI: 2.1e-05–0.59, *p* = 0.03) and gross total resection (adjusted OR = 320.6, 95% CI: 6.19–16,596, *p* = 0.004) prevailed as independent prognostic indicators (Fig. 7b).

A classification decision tree was constructed using a recursive partitioning algorithm to identify the most informative variable combinations in an integrated stratification model (Fig. 7c). Affirmingly, this algorithm selected stratification by tumour invasion, gross total resection, and molecular subgroup 1 as the most informative parameters in a hierarchical structure. Stratification based on this model yielded three overall risk groups we named “high”, “moderate” and “low” (Fig. 7c). This integrated model outperformed stratification approaches based on other currently established systems, also considering extended minimum follow-up time (Supplementary Fig. 6a–d). Additionally, investigating chromosomal alteration patterns of a previously published external series of aggressive and metastatic corticotroph PitNETs (*n* = 28)^29^ supported the role of subgroup 1 as a high-risk tumour subgroup (Supplementary Fig. 7).

### MagicTPiT classifier development

**Fig. 8:**
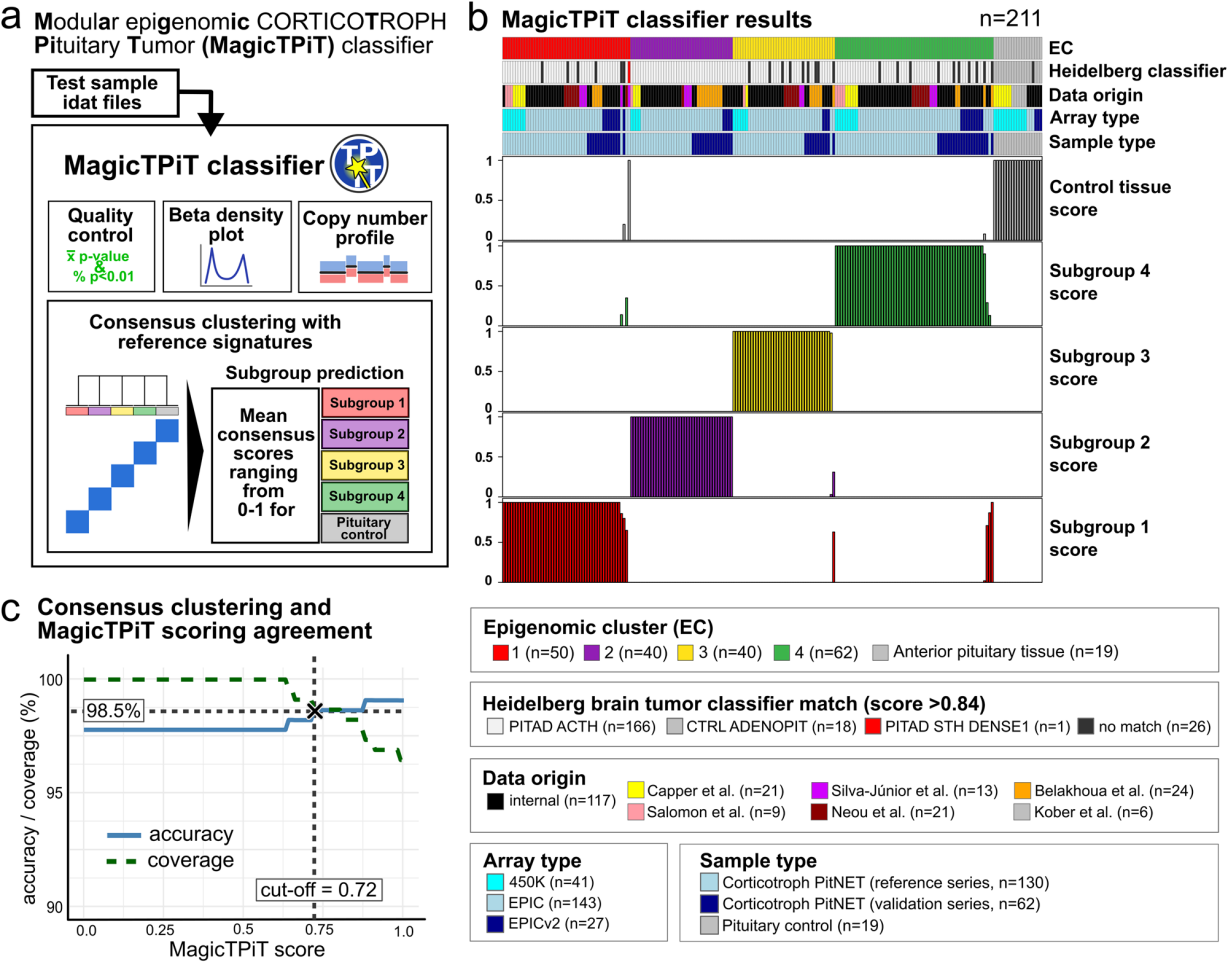
Development and validation of the MagicTPiT classifier. **a** The Modular Epigenomic CORTICOTROPH Pituitary Tumour (MagicTPiT) classifier was designed to process idat files of test samples. In parallel to calculating quality control parameters, including a beta-density plot and generating a copy number profile, each sample’s beta values are integrated into a dataset containing representative reference epigenomic signatures from the molecular subgroups (derived from the reference series) and anterior pituitary control tissue. Consensus clustering is performed and mean consensus scores are calculated for each test sample relative to the subgroups 1-4 and to control tissue. **b** All samples of the reference and validation series were scored using the MagicTPiT classifier. Sample origins and methylation array types did not evidently influence scoring results. **c** Sample subgroup assignments based on the original consensus clustering were compared to the MagicTPiT classifier scores. Using a cut-off of 0.72 to define a match, the classifier achieved 98.5% accuracy (concordance between original clustering and classifier assignment) and 98.5% coverage (percentage of samples with a score at or above this cut-off). Source data are provided as a Source Data file.

Given that currently available methylation-based classifiers do not distinguish between the molecular subgroups of corticotroph PitNETs, we developed an easy-to-use epigenomic classification tool based on representative tumour cluster signatures defined by the reference series of this study. An R Shiny application was designed to provide a user-friendly interface enabling the processing of user-provided raw DNA-methylation data to calculate scores for molecular subgroups 1-4 and pituitary control tissue. Additional modules were implemented to perform quality control, visualise beta-value densities and generate copy-number profiles. We named the resulting tool the Modular Epigenomic CORTICOTROPH Pituitary Tumour (MagicTPiT) classifier (Fig. 8a and Supplementary Fig. 8). Methylation data of all tumours of the reference series (*n* = 130) and validation series (*n* = 62) as well as anterior pituitary tissue (*n* = 19) were processed using the MagicTPiT classifier. We found scores highly consistent with the original tumour clusters (Fig. 8b, Fig. 1e and Supplementary Fig. 3c). Internal (*n* = 6) and external non-neoplastic anterior pituitary tissues (*n* = 13)^21,30^ all received scores of 1.0 for pituitary control tissue (Fig. 8b). Considering a cut-off of 0.72 for a molecular subgroup match yielded 98.5% coverage of samples and 98.5% accuracy in relation to the original tumour clusters (Fig. 8c). Further comparing the results of MagicTPiT with the Heidelberg classifier suggested that using both these tools in concert further improves diagnostic power and predictive certainty (Supplementary Fig. 9).

## Discussion

In this study, we identified four molecular subgroups of corticotroph tumours defined by specific epigenomic, transcriptomic and proteomic features. In brief, subgroup 1 primarily comprised large silent and functioning tumours with frequent copy-number alterations. Consistent with earlier reports linking genomic instability to aggressiveness^31,32^, this subgroup was associated with inferior clinical trajectories. Subgroup 2 tumours were mostly silent, copy number stable and were all found in female patients. Subgroup 3 tumours of this study correspond to previously described GATA3-expressing corticotroph PitNETs with gonadotroph gene expression signatures found in male and female patients^19^. Subgroup 4 tumours align with previously described *USP8*- or *USP48*-variant, mostly functioning microtumours^14,16,17,19,20^. Previously published work by Neou et al.^19^ and Ricklefs et al.^20^ suggested that corticotroph PitNETs comprise three instead of four molecular subgroups. Our results indicate that subgroup 2 tumours were underrepresented in these two studies and thus were not recognised as molecularly distinct. This subgroup was observed exclusively in females, which may indicate sex-specific biological mechanisms of tumourigenesis. SSTR1 expression was previously implicated in corticotroph PitNETs, with few studies reporting its increased expression in a subset of silent tumours^33,34^. We show that SSTR1 serves as a specific surrogate marker for subgroup 2.

According to the current WHO classification, corticotroph PitNETs are immunopositive for TPIT and lack SF1 and PIT1 expression; whereas multilineage PitNETs are recognised as a distinct tumour group^1,6^. We and others have previously reported frequent co-expression of PIT1 and SF1 in a subset of molecularly distinct PitNETs, which otherwise closely resemble densely granulated somatotroph tumours^19,35–37^. We included several corticotroph PitNETs with variable SF1 expression in this study (21/94; 22.3%). Of note, these tumours were purposefully selected, and the overall prevalence of such tumours is likely much lower in the general population. In line with findings by Asa et al.^38^, SF1-immunopositivity was seen only in a subset of tumour cells per case. We did not find a correlation of SF1 and the molecular subgroups 1-4, suggesting that SF1-expression does not associate with molecular distinctness in corticotroph PitNETs, unlike what has been reported for PitNETs related to the somatotroph lineage^35,37,39^.

We show that the three currently established WHO subtypes do not fully reflect the four molecular subgroups identified in this study. Of note, Crooke cell tumours -defined by CAM5.2 immunostainings- did not form a distinct molecular subgroup, and several included tumours exhibited ambiguous granulation patterns. These findings highlight limitations of the current histological subtyping standards. Whether refinement of histological granulation criteria or the incorporation of ultrastructural features may improve alignment with molecular subgroups remains to be determined. To enable identification of molecular subgroups in routine histopathology, we demonstrate the utility of SSTR1, GATA3, and SSTR5 immunohistochemistry as surrogate markers.

The molecular subgroups informed prognostic stratification when incorporated into an integrated model, which additionally considers evidence of tumour invasion and achievement of gross total resection. Interestingly, a Ki-67 index ≥3% did not emerge as a robust prognostic parameter in our analyses. This may indicate that an adjustment to this commonly used cut-off is warranted, or that the value of assessing proliferative activity should be reconsidered in the context of specific PitNET lineages and subgroups.

Our integrated stratification model was based on clinical trajectories defined by required treatment modalities and reported tumour aggressiveness, serving as a proxy for tumour behaviour and overall disease burden. It should be noted that this categorisation did not account for morbidity associated with endocrine dysfunction or the repeated surgical attempts sometimes required to detect small non-invasive tumours. Moreover, due to a lack of consistent reporting, therapy response and disease control could not contribute to the definitions of clinical trajectories in our study. We further acknowledge that confounders arise from variability in treatment decisions and surgical techniques across centres and that, also due to limited follow-up time in the series, further prospective studies will be required to determine the true prognostic value of the proposed integrated model.

Notably, some tumours lacking expression of SSTR1, GATA3, and SSTR5 did not align epigenetically with subgroup 1 in our series. Future studies should aim to clarify whether the use of surrogate markers alone provides sufficient value for prognostic stratification. To facilitate this, we provide the easy-to-use MagicTPiT classifier for methylation-based subgrouping of corticotroph PitNETs.

Several methodological considerations related to meta-analyses and the retrospective study design should also be acknowledged. While combining internal and external samples into larger pooled series enables broader data exploration and increases statistical power, it also introduces potential sources of bias due to heterogeneity in sample annotations across studies. Several relevant tumour features may have been impacted by interobserver variability and inconsistent definitions. For example, cavernous sinus invasion could not be defined consistently across studies using radiological or surgical criteria. It should also be noted that case selection was guided by the objectives of this study. For internal cases, tissue availability and intertumoural heterogeneity primarily determined inclusion, whereas external cases were subject to the selection criteria of their original publications. Consequently, the overall tumour characteristics and subgroup prevalences reported in this study should not be considered to represent real-world distributions.

## Methods

The work performed in this project complies with all relevant ethical regulations, and informed written consent was acquired from patients. Specifically, the use of surgery specimens and associated clinical data for research was conducted in accordance with local and national ethical standards and approved by the ethics committees of the respective medical institutions (UKE Hamburg: 25-300588-WF, PV4904; LMU Munich: 643-16; MSCNRIO Warsaw: 15/2021).

### Internal case series assembly

An internal case series comprising a total of 111 corticotroph PitNETs was aggregated. Inclusion required (neuro)pathological confirmation of diagnosis, and cases were purposefully selected to reflect a broad spectrum of clinicopathological features. For all internal cases, diagnoses were confirmed at the Institute of Neuropathology of the University Medical Centre Hamburg-Eppendorf based on epigenomic profiling and/or histopathological evaluation by at least two local board-certified (neuro)pathologists (MD, WS, JM, US, MG, JEN). A subset of the internal case series was previously published by Ricklefs et al.^20^ (*n* = 36), Dottermusch et al.^40^ (*n* = 4), Pękul et al.^41^ (*n* = 4), Rafiq et al.^42^ (*n* = 1), Sumislawski et al.^43^ (*n* = 1), and Albani et al.^27^ (*n* = 1). Further details are provided in Supplementary Data 1.

### Histology and immunohistochemistry

FFPE tissue samples were sectioned into 2 µm thick slices and stained for H&E and PAS according to standard laboratory protocols. Immunohistochemical stainings were performed on an automated staining machine (Ventana BenchMark XT, Roche Diagnostics, Mannheim, Germany). The following primary antibodies were used: TPIT (Atlas antibodies, AMAb91409, CL6251, MAB03695, 1:1000), PIT1 (Abcam, ab272639, Polyclonal, GR3446201-1, 1:1000), SF1 (Abcam, ab217317, EPR19744, GR3338943-4, 1:250), ACTH (Diagnostic BioSystems, RP045, Polyclonal, 395 T, 1:500), CAM5.2 (BD Biosciences, 345779, CAM5.2, 7058780, 1:1000), Ki-67 (Cell Marque, 275R-15, SP6, 1232104 A, 1:750), SSTR5 (Abcam, ab109495, UMB4, GR3222221-1, 1:200), SSTR1 (Invitrogen, 704008, 18H22L9, 2971844, 1:100), GATA3 (BioCare Medical, CM405A, L50-823, 102621 A, 1:75). Detection was performed with secondary antibodies and diaminobenzidine (DAB) as a chromogen using the OptiView or UltraView Universal DAB Detection Kit.

### Histological assessments and tumour subtyping

Internal samples were visually assessed for PAS staining intensities and immunopositivity for ACTH, SSTR1, SSTR5, GATA3, TPIT, PIT1, SF1, as well as Ki-67 by two board-certified neuropathologists (MD and JEN), independently. Stainings were scored according to the percentage of distinctly positively stained tumour cells as follows: 0, 1, 3, 5, 10, 20, and subsequent increments of 10 up to 100. Ki-67-indices were estimated as whole-number percentages for the entire tumour. For PAS, ACTH and SSTR1, diffuse cytoplasmic tumour staining was considered positive. For SSTR5, only membranous tumour staining was considered positive. For GATA3, TPIT, PIT1, SF1 and Ki-67, only nuclear tumour staining was considered positive. Crooke’s changes were quantified based on the percentage of tumour cells showing distinctive ring-like perinuclear CAM5.2 staining.

Histological subtyping of corticotroph PitNETs was based on predefined criteria using the mean scorings of both observers. Sparse granulation was attributed to tumours with <33% PAS- and <33% ACTH-positive tumour cells. Dense granulation was attributed to tumours with >66 % PAS- and >66% ACTH- positive tumour cells. All tumours with ≥50% Crooke tumour cells were designated as Crooke cell tumours. All remaining tumours were categorised as showing ambiguous granulation patterns.

Interobserver agreement for histological subtyping was quantified using the Jaccard similarity coefficient, calculated as the ratio of intersections to the unions of subtype assignments based on individual observer scorings.

### Data and image processing

Computational analyses were conducted in R software (v4.3.3)^44^ using the stats (v4.3.3) and irr R package (v0.84.1). Graphs were generated with ggplot2 (v3.5.1) and plotly R package (v4.10.4). Histological slides were digitised with a Hamamatsu NanoZoomer S20MD to obtain representative tissue images and exported using NDP.view software (v2.7.52). Tissue images were white-balanced using Adobe Photoshop software (v24.7.0). All figures containing images and graphs were processed using Inkscape software (v1.2.1).

### Raw DNA methylation data acquisition

DNA was isolated from FFPE tumour tissue using the Maxwell FFPE Plus DNA Kit (Promega). Around 100–500 ng of DNA was bisulfite-converted using the EZ DNA Methylation Kit (Zymo Research). The DNA Clean & Concentrator-5 kit (Zymo Research) and the Infinium HD FFPE DNA Restore Kit (Illumina) were used to clean and restore the converted DNA. Finally, the Illumina Infinium MethylationEPIC and Infinium MethylationEPIC v2.0 BeadChip arrays were used to quantify the methylation statuses of CpG sites on an iScan device (Illumina).

### Raw protein data acquisition

FFPE tumour tissue was collected and processed for proteome analysis. Protein concentrations were determined using the Pierce BCA Protein assay kit (Thermo Fisher), and 10 µg of protein per sample were processed using a semi-automated SP3 protocol in 96-well LoBind plates (Eppendorf). Disulphide bonds were reduced in 10 mM dithiothreitol and alkylated with 20 mM iodoacetamide. Proteins were captured on carboxylate-modified magnetic beads (E3/E7, Cytiva Sera-Mag) at 50% ACN, washed with 80% ethanol and 100% ACN, and digested overnight with trypsin (Promega, 1:100 enzyme: protein) in 100 mM AmBiCa. Digestion was stopped with 1% trifluoroacetic acid, and supernatants were collected for LC-MS/MS analysis. Peptides were separated on a 25 cm C18 column (Aurora Ultimate, IonOptics) using a 70-min gradient (3-34% ACN) on a Vanquish neo UHPLC system (Thermo Fisher) with online desalting via a PepMap Neo trap cartridge. MS/MS data were acquired in data-independent acquisition (DIA) mode on an Exploris 480 mass spectrometer (Thermo Fisher) with HCD fragmentation. LC-MS/MS data were searched with the CHIMERYS DIA algorithm in Proteome Discoverer (v3.1.0.638, Thermo Fisher Scientific) against a reviewed human Swissprot database using INFERYS 3.0 fragmentation as a prediction model. Carbamidomethylation for cysteine residues was set as fixed, and methionine oxidation as a variable modification. The maximum number of missing tryptic cleavage was set to one. Peptides of 7–30 amino acids were retained at FDR < 0.01. Quantification was performed based on fragment ions and normalised to total peptide amount. Protein count data were log2-transformed and median-centred sample-wise.

### Integration of external cases and molecular data

The internal series of 111 cases was augmented with sample annotations and molecular data of 159 externally diagnosed and previously published corticotroph PitNETs, amounting to a total of 270 cases. External samples were compiled from studies which provided publicly available and accessible molecular data, including raw epigenomic data and processed transcriptomic and proteomic data. To mitigate batch effects, only studies containing both functioning and silent tumours were included. Sample inclusion further required histopathological confirmation of corticotroph tumour diagnosis as reported by the respective studies. Further details are provided in Supplementary Data 2.

Raw methylation array data (idat files) derived from Illumina 450K or EPIC arrays by the following studies were obtained from publicly available deposits: Neou et al. (E-MTAB-7762; *n* = 21)^19^, Belakhoua et al. (GSE283928; *n* = 24)^28^, Silva-Júnior et al. (GSE207937; *n* = 13)^24^, Salomon et al. (GSE147548; *n* = 9)^22^, and Capper et al. (GSE109381; *n* = 14)^21^. For all included samples, it was verified that beta-value distributions were not aberrant, and that they were not assigned to a methylation class incompatible with PitNET diagnosis via the Heidelberg classifier v12.8. Internally and externally derived idat files were processed together, as described in the subsequent section “DNA methylation data processing”.

Externally processed gene expression data derived from high-throughput RNA sequencing were obtained from publicly available deposits: Bujko et al. (GSE132982; *n* = 20)^17^, Zhang et al. (10.6084/m9.figshare.21340161 (v4); *n* = 44)^23^, Neou et al. (E-MTAB-7768; *n* = 35; 21 of which were also in the epigenomic dataset)^19^, Silva-Júnior et al. (10.5281/zenodo.6565109 (v1); *n* = 13; all of which were also in the epigenomic dataset)^24^. Gene expression matrices derived from these different studies were log2 transformed and merged while correcting for study-related batch effects using ComBat of the sva R package (v3.50.0).

Externally processed protein count data derived from liquid chromatography-tandem mass spectrometry (LC-MS/MS) of the 44 corticotroph PitNETs previously published by Zhang et al^23^ was obtained via 10.6084/m9.figshare.21340161. These external protein data were log2-transformed and merged with the internal cases, excluding proteins with less than 50% valid values. Study-related batch effects were corrected using ComBat of the sva R package (v3.50.0). Missing values were imputed using the impute R package (v1.76.0) based on the K-nearest neighbour (KNN) algorithm with nearest neighbours set to five.

### Study design and series assignment

The 270 samples were assigned to either a reference or a validation series based on the time of sample availability. The reference series (*n* = 208) comprised initially available cases and was used for primary analyses. Subsequently, the validation series (*n* = 62) was compiled. Allocation of samples was therefore determined by chronological inclusion and was not influenced by clinical, pathological, or molecular characteristics. Both series were processed and analysed using the same protocols, as described below.

### DNA methylation data processing

Raw methylation array data (idat files) were processed using the minfi R package (v1.48.0). Probes on sex chromosomes, probes with a detection *p*-value ≥ 0.01, probes with SNPs at the CpG site, and cross-reactive probes were excluded. When combining different types of arrays, probes, which were not consistently represented across the EPICv2, EPIC and 450K arrays, were excluded. Methylation-based classification using the Heidelberg brain tumour methylation classifier was performed using the mnp R packages versions 11b4, 12.6 and 12.8^21^.

### Cluster analyses

To calculate the goodness of clustering measure, the gap statistic was performed using the clusGap function of the cluster R package (v2.1.6). The optimal number of clusters was determined using the criterion of Tibshirani et al.^45^.

Hierarchical consensus cluster analyses were performed using the ConsensusClusterPlus R package (v1.66.0) based on euclidean distance and ward.D2 linkage. Cluster analyses were based on beta-values of the top 2500 variant CpGs for DNA methylation data, on expression values of the top 250 variant genes for RNA data and on abundance values of the top 250 proteins for proteome data. Uniform Manifold Approximation and Projection (UMAP) transformation and plotting were performed using the umap R package (v0.2.10.0).

### Cumulative copy number analyses

Copy number profiles (CNPs) and overall CNP noise levels were calculated using the conumee R package (v1.36.0). Cumulative CNPs (CCNPs) were calculated using the GenVisR R package (v1.34.0). To reduce technical CCNP noise, short genomic segments <15 Mb length were excluded from cumulative analyses and the cut-off to determine gains and losses was set to one fourth of the overall noise level of each individual sample.

### Gene set enrichment analyses (GSEA)

GSEA was performed using the clusterProfiler R package (v4.10.0) based on the Gene Ontology (GO) Biological Process (BP) database. Genes or proteins were ranked by median fold changes obtained when comparing one molecular subgroup to the other three. Gene set size ranges were set to 10–1000. GO terms were reduced for redundancy using the clusterProfiler simplify function with a cut-off of 0.7 (Wang similarity measure), thereby preserving more significant terms. Significantly enriched gene sets with *p*-values < 0.05 were compiled and adjusted for multiple comparisons using Bonferroni correction.

Single-sample GSEA was performed using the GSVA package (v1.42.0). The gene sets “stemness”, “synapse assembly” and “secretory vesicle” were obtained from the Harmonizome 3.0 database^46^. The gene set “gonadotrophs” was compiled using on the top 20 significantly upregulated genes of gonadotroph PitNETs compared to non-gonadotroph PitNET types in the RNA dataset published by Neou et al. (E-MTAB-7768)^19^.

### Clinicopathological data integration

Tumour invasion was assessed based on evidence of cavernous sinus infiltration. When available, a Knosp grade >2 was considered invasive; otherwise, invasion was inferred from explicit radiological or intraoperative descriptions of tumour infiltration or extension into the cavernous sinus.

Tumour hormonal activity was classified as silent when neither biochemical nor clinical evidence of hypercortisolism was present. Subclinical CD was defined as biochemical evidence of hypercortisolism in the absence of overt clinical manifestations of Cushing’s syndrome. Overt CD was defined by the presence of both biochemical evidence of hypercortisolism and clinical features consistent with Cushing’s syndrome (e.g., central weight gain, facial plethora, proximal myopathy, hypertension, diabetes mellitus, and/or skin changes such as easy bruising or striae). For internal cases, biochemical hypercortisolism was defined by at least one of the following criteria: elevated 24-hour urinary free cortisol (> assay-specific reference range), elevated late-night salivary cortisol (> 145 ng/dl), or elevated serum cortisol after a 1 mg overnight dexamethasone suppression test (> 1.8 µg/dL, 50 nmol/L).

Proliferation assessed by Ki-67 index was categorised as ≥3% or <3%. When available, these categories were derived from Ki-67 indices reported as continuous variables. One study reported Ki-67 index categories as >3% and ≤3%^23^. To enable data integration, these categories were relabelled as ≥3% and <3%, respectively.

### *USP8* and *USP48* variant analyses

*USP8* and *USP48* hotspot regions were Sanger sequenced using primers previously described^47^ (*USP8* forward: TTCTTGACCCAATCACTGG, reverse: GAAATTTCCAACTCCCTGACAC; *USP48* forward: GCCCCGCTAAAGAATAAACA, reverse: TGCCTGCTATAATCCTGGAAA). PCR was performed using 50 ng genomic DNA in 25 μL reactions containing 1.5 mM MgCl₂, 0.2 μM of each primer, 200 μM dNTPs, and 1.25 U Dream Taq DNA Polymerase (Thermo Fisher), with 30 cycles of 94 °C for 20 s, 58 °C for 30 s, and 72 °C for 30 s. Amplicons were sequenced by Eurofins Genomics using the Mix2Seq Kit NightXpress.

For internal and external cases, *USP8* variants were defined as hotspot alterations in exon 14, comprising in-frame deletions (p.Ser718del and p.Pro720del) and missense variants, which included p.Ser718Pro, p.Ser718Phe, p.Ser719Pro, p.Pro720Glu, and p.Pro720Arg. *USP48* variants were defined as hotspot missense alterations in exon 10, which included p.Met415Val and p.Met415Ile.

### Prognostic stratification

Cases were grouped according to clinical trajectories defined as “initial remission” (no second intervention after initial tumour resection, no biochemical or imaging evidence of residual disease after at least 6 months of follow-up), “repeated surgeries / persistent or recurrent disease” (more than one tumour resection and/or evidence of disease at last follow-up), or “multimodal therapy / aggressive disease” (additional interventions after tumour resection in the form of radiotherapy/chemotherapy, and/or cases defined as aggressive in the original publications). For the groups “initial remission” and “repeated surgeries / persistent or recurrent disease” a minimum follow-up of 6 months was required to assess disease course and exclude occurrence of criteria defining the group “multimodal therapy / aggressive disease”.

Ordinal logistic regression was performed using the R ordinal package (v2024.12-4). Univariable cumulative link models were used to assess associations between clinicopathological and molecular variables and ordered clinical trajectories, with *p*-values derived from likelihood ratio tests. Predictors were treated as categorical variables with predefined reference levels, and missing data were handled by complete-case analyses per model. Additionally, a multivariable model including all selected covariates was fitted to assess independent associations.

A classification tree was fitted using the R rpart package (v4.1.23). Initial model complexity was controlled using a complexity parameter of 0.1. The final tree was selected using cost-complexity pruning based on the minimum cross-validated relative error. To simplify interpretation, terminal node (leaf) class probabilities were extracted and aggregated by averaging probabilities of nodes sharing the same dominant prediction class.

### MagicTPiT classifier development and deployment

To provide a user-friendly tool for classifying corticotroph PitNETs based on DNA methylation data, the Modular Epigenomic CORTICOTROPH Pituitary Tumour (MagicTPiT) classifier was developed using the R shiny package (v.1.10.0). The classifier was packaged into a standalone Windows desktop application based on the DesktopDeployR framework by W. Lee Pang (https://github.com/wleepang/DesktopDeployR). Inno Setup is a free installer for Windows programmes by Jordan Russell and Martijn Laan (https://jrsoftware.org/isinfo.php) and was used to compile an easily installable version of the software. The MagicTPiT classifier allows users to process EPICv2, EPIC or 450K idats of corticotroph PitNETs and perform consensus clustering together with reference signatures of the molecular subgroups 1–4 and anterior pituitary control tissue.

For building a set of representative reference signatures to serve as the basis of consensus clustering, hierarchical clustering based on beta-values was performed on the subgroups of the tumour reference series including anterior pituitary control tissue samples (*n* = 6) derived from Kober et al.^30^. To exclude signature outliers, four samples were removed that showed incongruent subgroup affiliations when comparing consensus clustering with UMAP results. Additionally, 173,462 CpG sites were removed in which at least one sample per subgroup deviated by ≥0.4 from the subgroup median beta value. Each subgroup was then partitioned into six clusters. To represent each cluster, beta values of all members were averaged, resulting in a reduced set of representative sample profiles. For each CpG site, an F-test was performed to test for methylation differences across the representative subgroup signatures. Each CpG was assigned to the subgroup in which the beta-values deviated most strongly from the overall mean. CpG not represented across 450K, EPIC and EPICv2 arrays were removed. The 100 most significant CpGs for each subgroup were selected, yielding a balanced dataset comprising 30 representative reference signature samples and 500 discriminatory CpG sites. Following consensus clustering of test samples with these reference signatures, prediction scores were calculated as the mean consensus scores of the test sample and all samples of each subgroup. Additional modules were included to perform quality control, display beta value densities, and generate copy number profiles, in accordance with the methods described in this manuscript.

### Use of generative AI in manuscript preparation

For the preparation of this manuscript, ChatGPT (OpenAI) was used in order to assist in refining language and improving sentence readability. All AI-generated content was reviewed and edited by the authors, who take full responsibility for the article's final content.

### Statistics & reproducibility

In this study, no statistical method was used to predetermine sample sizes, as case selection was guided and limited by the availability of suitable internal and external cases. Samples with insufficient confirmation of PitNET diagnosis due to inadequate histopathology or poor molecular data quality were excluded from the study. For measurements that required human judgment, investigators were blinded to molecular subgroup allocations of samples.

### Reporting summary

Further information on research design is available in the Nature Portfolio Reporting Summary linked to this article.

## Supplementary information

Supplementary Information

Description of Additional Supplementary File

Supplementary Data 1

Supplementary Data 2

Supplementary Data 3

Supplementary Data 4

Supplementary Data 5

Supplementary Data 6

Supplementary Data 7

Reporting Summary

Transparent Peer Review file

## Source data

Source Data

## Data Availability

External publicly available epigenome, transcriptome and proteome data used in this study are available in Gene Expression Omnibus under the accession codes. GSE132982^17^, GSE109381^21^, GSE147548^22^, GSE207937^24^, GSE283928^28^, in ArrayExpress under the accession codes. E-MTAB-7768^19^, E-MTAB-7762^19^, in Figshare [10.6084/m9.figshare.21340161] (v4)^23^ and in Zenodo [10.5281/zenodo.6565110] (v1)^24^. Raw and processed epigenome and proteome data generated in this study are available in ArrayExpress (E-MTAB-16589 [https://www.ebi.ac.uk/biostudies/arrayexpress/studies/E-MTAB-16589] and PRIDE (PXD073952; [https://www.ebi.ac.uk/pride/archive/projects/PXD073952], respectively. Remaining data are available within this article, the Supplementary Information and the Source Data file. Source data are provided with this paper.

## Code availability

The MagicTPiT classifier and its code have been made available as an R package on GitHub [https://github.com/MatthiasDottermusch/MagicTPiT]. The installer for the standalone Windows desktop version and all required installation instructions are also available via this link under the Releases section.
