# Supplementary material for: Multi-omics integration unravels four molecular subgroups of corticotroph pituitary neuroendocrine tumours with distinct clinicopathological features
Source: Nat Commun. 2026 Aug 4;17:7777. doi: 10.1038/s41467-026-76292-y (PMC13438112; doi:10.1038/s41467-026-76292-y)
Supplement: Supplementary file 1 [file 41467_2026_76292_MOESM10_ESM.pdf]

nature portfolio

Corresponding author(s): Matthias Dottermusch, Julia Neumann

Last updated by author(s): Jul 5, 2026

# Reporting Summary

Nature Portfolio wishes to improve the reproducibility of the work that we publish. This form provides structure for consistency and transparency in reporting. For further information on Nature Portfolio policies, see our Editorial Policies and the Editorial Policy Checklist.

## Statistics

For all statistical analyses, confirm that the following items are present in the figure legend, table legend, main text, or Methods section.

| n/a | Confirmed | |
|---|---|---|
| ☐ | ☒ | The exact sample size (*n*) for each experimental group/condition, given as a discrete number and unit of measurement |
| ☐ | ☒ | A statement on whether measurements were taken from distinct samples or whether the same sample was measured repeatedly |
| ☐ | ☒ | The statistical test(s) used AND whether they are one- or two-sided<br>*Only common tests should be described solely by name; describe more complex techniques in the Methods section.* |
| ☐ | ☒ | A description of all covariates tested |
| ☐ | ☒ | A description of any assumptions or corrections, such as tests of normality and adjustment for multiple comparisons |
| ☐ | ☒ | A full description of the statistical parameters including central tendency (e.g. means) or other basic estimates (e.g. regression coefficient) AND variation (e.g. standard deviation) or associated estimates of uncertainty (e.g. confidence intervals) |
| ☐ | ☒ | For null hypothesis testing, the test statistic (e.g. *F*, *t*, *r*) with confidence intervals, effect sizes, degrees of freedom and *P* value noted<br>*Give P values as exact values whenever suitable.* |
| ☒ | ☐ | For Bayesian analysis, information on the choice of priors and Markov chain Monte Carlo settings |
| ☐ | ☒ | For hierarchical and complex designs, identification of the appropriate level for tests and full reporting of outcomes |
| ☐ | ☒ | Estimates of effect sizes (e.g. Cohen's *d*, Pearson's *r*), indicating how they were calculated |

*Our web collection on statistics for biologists contains articles on many of the points above.*

## Software and code

Policy information about availability of computer code

| | |
|---|---|
| Data collection | Methylation data were generated on the Illumina iScan system using iScan Control Software (v3.4.8).<br>Proteome data were generated using Proteome Discoverer (v3.1.0.638) with the CHIMERYS and INFERYS 3.0 algorithm. |
| Data analysis | Computational analyses were conducted in R software (v4.3.3) using the stats R package (v4.3.3). Graphs were generated with ggplot2 R package (v3.5.1) and plotly R package (v4.10.4). Raw methylation array data (idat files) were processed using the minfi R package (v1.48.0). Methylation-based classification using the Heidelberg brain tumor methylation classifier was performed using mnp R packages versions 11b4, 12.6 and 12.8. Copy number profiles (CNPs) and overall CNP noise levels were calculated using the conumee R package (v1.36.0). Cumulative CNPs (CCNPs) were calculated using the GenVisR R package (v1.34.0). Study-related batch effects were corrected with ComBat using the sva R package (v3.50.0). Proteome data missing values were imputed using the impute R package (v1.76.0). Gap statistic was performed using the clusGap function of the cluster R package (v2.1.6). Hierarchical consensus cluster analyses were performed using the ConsensusClusterPlus R package (v1.66.0). Uniform Manifold Approximation and Projection (UMAP) transformation and plotting were performed using the umap R package (v0.2.10.0). Gene set enrichment analysis was performed using the clusterProfiler R package (v4.10.0) based on the gene ontology (GO) biological process (BP) database. Single-sample GSEA was performed using the GSVA package (v1.42.0). Cramer's V was computed using the assocstats-function of the vcd R package (v1.4-13). Cohen's kappa was computed using the irr R package (v0.84.1). Ordinal logistic regression was performed using the R ordinal package (v2024.12-4). Classification trees were fitted using the rpart R package (v4.1.23). |

For manuscripts utilizing custom algorithms or software that are central to the research but not yet described in published literature, software must be made available to editors and reviewers. We strongly encourage code deposition in a community repository (e.g. GitHub). See the Nature Portfolio guidelines for submitting code & software for further information.

# Data

Policy information about availability of data

All manuscripts must include a data availability statement. This statement should provide the following information, where applicable:

- Accession codes, unique identifiers, or web links for publicly available datasets
- A description of any restrictions on data availability
- For clinical datasets or third party data, please ensure that the statement adheres to our policy

Raw and processed epigenome and proteome data generated in this study were deposited in the publicly-accessible repositories ArrayExpress (https://www.ebi.ac.uk/biostudies/arrayexpress/studies/E-MTAB-16589) and PRIDE (https://www.ebi.ac.uk/pride/archive/projects/PXD073952), respectively. The MagicTPiT classifier and its code has been made available as an R package and standalone application via github (https://github.com/MatthiasDottermusch/MagicTPiT).

# Research involving human participants, their data, or biological material

Policy information about studies with human participants or human data. See also policy information about sex, gender (identity/presentation), and sexual orientation and race, ethnicity and racism.

| | |
|---|---|
| Reporting on sex and gender | Sex was assessed as a biological attribute based on available patient data or the original sample annotations. In total the study included data from 202 females and 68 males. |
| Reporting on race, ethnicity, or other socially relevant groupings | Race, ethnicity, or other socially relevant groupings were not considered for sample inclusion, nor were they assessed in this study. |
| Population characteristics | The investigated case series comprised patients with histopathologically confirmed diagnosis of corticotroph pituitary neuroendocrine tumor/adenoma, derived from internal and publicly available external datasets. Characteristics of the case series are provided in Supplementary Data 1. |
| Recruitment | No participants were recruited for the purpose of conducting this study. |
| Ethics oversight | The work performed in this project complies with all relevant ethical regulations and informed consent was acquired from patients. Specifically, the use of surgery specimens and associated clinical data for research were conducted in accordance with local and national ethical standards and approved by the ethics committees of the respective medical institutions (University Medical Center Hamburg-Eppendorf: 25-300588-WF, PV4904; Ludwig-Maximilian University Munich: 643-16; Maria Skłodowska-Curie National Research Institute of Oncology Warsaw: 15/2021). |

Note that full information on the approval of the study protocol must also be provided in the manuscript.

# Field-specific reporting

Please select the one below that is the best fit for your research. If you are not sure, read the appropriate sections before making your selection.

☒ Life sciences ☐ Behavioural & social sciences ☐ Ecological, evolutionary & environmental sciences

For a reference copy of the document with all sections, see nature.com/documents/nr-reporting-summary-flat.pdf

# Life sciences study design

All studies must disclose on these points even when the disclosure is negative.

| | |
|---|---|
| Sample size | The study included all available samples meeting the defined inclusion criteria. No formal sample-size calculation was performed; inclusion was limited by availability and data quality of internal and external datasets. |
| Data exclusions | Samples were excluded if they failed quality control, as indicated by aberrant beta-value distributions, or if their methylation class assignment via the Heidelberg classifier v12.8 was incompatible with pituitary neuroendocrine tumor/adenoma diagnosis. |
| Replication | Relevant findings from the reference series were replicated in an independent validation series. |
| Randomization | Cases were organized into a reference and a validation series. Assignment was based on the order and timing of sample collection, rather than randomization. |
| Blinding | For measurements that required human judgment, investigators were blinded to group allocation. |

# Reporting for specific materials, systems and methods

We require information from authors about some types of materials, experimental systems and methods used in many studies. Here, indicate whether each material, system or method listed is relevant to your study. If you are not sure if a list item applies to your research, read the appropriate section before selecting a response.

## Materials & experimental systems

| n/a | Involved in the study |
|---|---|
| ☐ | ☒ Antibodies |
| ☒ | ☐ Eukaryotic cell lines |
| ☒ | ☐ Palaeontology and archaeology |
| ☒ | ☐ Animals and other organisms |
| ☒ | ☐ Clinical data |
| ☒ | ☐ Dual use research of concern |
| ☒ | ☐ Plants |

## Methods

| n/a | Involved in the study |
|---|---|
| ☒ | ☐ ChIP-seq |
| ☒ | ☐ Flow cytometry |
| ☒ | ☐ MRI-based neuroimaging |

# Antibodies

| | |
|---|---|
| Antibodies used | ACTH (Supplier: Diagnostic BioSystems, Cat. No.: RP045, Polyclonal, LOT: 395T, 1:500)<br>CAM5.2 (Supplier: BD Biosciences, Cat. No.: 345779, Clone: CAM5.2, LOT: 7058780, 1:1000)<br>GATA3 (Supplier: BioCare Medical, Cat. No.: CM405A, Clone: L50-823, LOT: 102621A, 1:75)<br>Ki-67 (Supplier: Cell Marque, Cat. No.: 275R-15, Clone: SP6, LOT: 1232104A, 1:750)<br>PIT1 (Supplier: Abcam, Cat. No.: ab272639, Polyclonal, LOT: GR3446201-1, 1:1000)<br>SF1 (Supplier: Abcam, Cat. No.: ab217317, Clone: EPR19744, LOT: GR3338943-4, 1:250)<br>SSTR1 (Supplier: Invitrogen, Cat. No.: 704008, Clone: 18H22L9, LOT: 2971844, 1:100)<br>SSTR5 (Supplier: Abcam, Cat. No.: ab109495, Clone: UMB4, LOT: GR3222221-1, 1:200)<br>TPIT (Supplier: Atlas antibodies, Cat. No.: AMAb91409, Clone: CL6251, LOT: MAB03695, 1:1000) |
| Validation | Sensitivity and specificity of all antibodies were validated by board-certified neuropathologists.<br>Technical success of immunostainings were confirmed by evaluation of appropriate positive and negative tissue controls. |

# Plants

| | |
|---|---|
| Seed stocks | No plants were used in this study. |
| Novel plant genotypes | No plants were used in this study. |
| Authentication | No plants were used in this study. |
