# Supplementary material for: Multi-omics integration unravels four molecular subgroups of corticotroph pituitary neuroendocrine tumours with distinct clinicopathological features
Source: Nat Commun. 2026 Aug 4;17:7777. doi: 10.1038/s41467-026-76292-y (PMC13438112; doi:10.1038/s41467-026-76292-y)
Supplement: Supplementary file 2 [file 41467_2026_76292_MOESM11_ESM.pdf]

nature portfolio

Peer Review File

Multi-omics Integration Unravels Four Molecular Subgroups of Corticotroph Pituitary Neuroendocrine Tumours with Distinct Clinicopathological Features

Corresponding Author: Dr Matthias Dottermusch

This file contains all reviewer reports in order by version, followed by all author rebuttals in order by version.

Version 0:

Reviewer comments:

Reviewer #1

(Remarks to the Author)
The authors present a multi-omic analysis of 270 corticotroph PitNETs, integrating epigenomic, transcriptomic, proteomic, and histopathologic data. In their analysis, the authors identify 4 distinct molecular subgroups of corticotroph PitNETs. Subgroup 1 contained mixed clinical presentation of both silent and overt Cushing disease, but was defined by numerous copy number alterations compared to the other subgroups. Subgroups 2 and 3 are mainly comprised of clinically silent corticotroph PitNETs and were distinguished by SSTR1 expression in subgroup 2 and GATA3 expression in subgroup 3. Subgroup 4 comprised of mainly overt Cushing disease presentation, along with increased expression of SSTR5. Additionally, the authors developed a methylation classifier for others to stratify specimens into their reported schema.

Based on the evidence as presented, the presence of 4 molecular groups is convincing, but the clinical significance of these groups is difficult to ascertain, as acknowledged by the authors. In part this is due to the clinical metadata associated with these samples, which is of course important when associating subgroups with clinical significance.

Major comments:
1. Can the authors clarify what clinical factors were used to determine “overt Cushing”? In the 6 cases of “subclinical Cushing” what led to the discovery of biochemical hypercortisolism in these patients? How was hypercortisolism determined in both “subclinical Cushing” and “overt Cushing” patients?

2. In Supplementary File 1, it seems that some of the tumors used in the series are from reoperation cases or metastasis resections. These tumors should be excluded or analyzed separately as comparing metastases or tumors that have undergone adjuvant therapy/experienced recurrence may introducing confounding genomic profiles when comparing to primary tumors.

3. Outcomes regarding surgical resection should be reported if available, especially for cases involving cavernous sinus involvement or tumors that are adjacent to the cavernous sinus. In these cases, was the medial wall of the cavernous sinus resected? This is important to specify as remission risk can be reduced by medial cavernous sinus wall resection in appropriate cases

4. The authors’ clinical data is limited based on lack of follow up. There are many patients within their own series that do not have any follow-up documented, yet have clinical data completed (i.e. Patient 25 and 26). However, how can the authors know if the patients truly do not have any recurrence, adjuvant therapies, metastasis, etc. if there is no follow up to base these conclusions on? Additionally, there are only 32 patients that have >15 months follow up. Given that recurrence in Cushing disease commonly occurs > 15 months following initial intervention, the current follow-up definition of 6 months as “single-surgery controlled” is not sufficient to accounts for the majority of cases that recur at longer follow up. This also affects the authors ability to characterize tumors as “high”, “moderate”, and “low” risk effectively since this outcome is based on whether tumors recur at a 6 month interval. Is there an opportunity for the authors to supplement the existing data with data for patients from their own institution at least?

5. When determining risk stratification, the authors investigated the predictive value of WHO classification, Trouillas

classification, and molecular subgroups. However, the authors could additionally investigate a univariate analysis followed by a multivariate analysis of all clinical and molecular features to determine which variables are predictive of disease outcomes in order to capture other variables such as gender, age, preoperative ACTH/cortisol levels, etc.

6. On Page 19, “Incorporation of corticotroph molecular subgroups into currently established prognostication frameworks”: how many samples were used to generate this prognostic data and classification tree? Ideally, only samples that have the full complement of data (i.e. no missing data in Supplementary File 1) should be included for this analysis. Please indicate in Supplementary File 1 which samples were used to create the classification decision tree.

Minor comments:
Page 11, Line 2: How was it decided which samples would be included in the reference series versus the validation series?

Page 20, Lines 17-18: Should this be 98.5% (not 0.985%)?

(Remarks on code availability)

Reviewer #2

(Remarks to the Author)
Dear Authors,

I have read your submitted manuscript entitled "Integrated Multi-omics Analyses Classify Corticotroph Pituitary Neuroendocrine Tumors into Four Molecular Subgroups with Distinct Clinicopathological Features" carefully and I have to make the following comments:
1. The idea of finding any differences at the molecular level among the corticotroph PitNETs is interesting, in particular with respect to the variable biological behavior of this group of PitNETs. However, I would like to ask you to elaborate more on the advantages of your proposed categorization in these four molecular subgroups and discuss about its superiority compared to the existing subgrouping in the current WHO Classification. You conclude in the results section "In summary, histomorphological features mainly differentiated subgroups 1 and 4 from subgroups 2 and 3, yet they did not reliably distinguish among all four molecular subgroups." Why should your proposed subgrouping be favoured over the existing classification?
2. You describe various experimental procedures that you carried out but in the way that they are presented they may confuse the reader. For example, you use at the beginning for the initial experiments a number of cases then you add several other numbers of cases (either internal or external) and at the end one tries to identify what belongs to what. Therefore, I would suggest amending your methods section to be better organized, thus avoiding any perplexity.
3. As far as immunostaining assessment and histological subtyping are concerned, the method that you described has a high degree of subjectivity; it depends on the "observer's eye". I do not think that it is possible for the neuro-(pathologist) to recognize small differences, like 66% and 67%. However, this is of paramount significance, as in the first case the tumor will be considered of intermediate positivity whereas in the second of abundant positivity for the corresponding marker. In order to solve this issue, I would suggest to use a more objective approach, for example through the use of an automated counting apparatus. Another option would be to examine interobserver variability and make the appropriate analytical adjustments.
4. I would like to kindly ask you to explain the controversial results, on the one hand the Ki-67 (a well established and widely used proliferation marker) is more abundant in group 4 tumors, which on the other hand these are the ones that have the most favourable prognosis. We must consider that this marker is used as integral part of the grading of some tumors, in particular the neuroendocrine neoplasms; the higher the Ki-67 expression the higher the grade of the tumor.
5. Regarding the included images, acquired from the performed histochemical and immunohistochemical stains, these should be improved and be replaced with others of higher magnifications, where one can clearly identify the various staining patterns (i.e., membranous, cytoplasmic and nuclear).
6. Further, in the Figure 4.: B-E should be replaced by C-F, G-J by I-L, L-O by O-R. Similarly the G and H and the L and M do not correspond well to what it is written in the relevant part of the figure legend. Furthermore, in Figure 5 there are different numbers of cohorts (e.g., patient sex: 270 cases, clinical presentation: 236 cases, tumor size: 244 cases). I do not really understand why there are these differences in the cohorts, and I could not find the explanation of these differences anywhere within the text.
Thank you in advance for considering my suggestions, which I hope will contribute to the improvement of the manuscript.

Kind regards,

The Reviewer

(Remarks on code availability)

Version 1:

Reviewer comments:

Reviewer #1

(Remarks to the Author)
The authors have now revised their manuscript and addressed my criticisms satisfactorily. Ultimately, although still with limitations regarding clinical significance, the manuscript is of interest. Notably, of the 4 molecular groups identified, only one is of apparent clinical significance (molecular group 1). The remaining groups may later be discovered to be of some importance, as well, but this manuscript provides, at minimum, basis to further investigate group 1 tumors in future studies.

(Remarks on code availability)

Reviewer #2

(Remarks to the Author)
Dear Authors,

Thank you for amending and improving your manuscript according to my comments.
I am satisfied of the final version of your paper and I recommend its acceptance and publication on the journal "Nature Communications".

Kind regards,

The Reviewer

(Remarks on code availability)

Open Access This Peer Review File is licensed under a Creative Commons Attribution 4.0 International License, which permits use, sharing, adaptation, distribution and reproduction in any medium or format, as long as you give appropriate credit to the original author(s) and the source, provide a link to the Creative Commons license, and indicate if changes were made.
In cases where reviewers are anonymous, credit should be given to 'Anonymous Referee' and the source.
The images or other third party material in this Peer Review File are included in the article's Creative Commons license, unless indicated otherwise in a credit line to the material. If material is not included in the article's Creative Commons license and your intended use is not permitted by statutory regulation or exceeds the permitted use, you will need to obtain permission directly from the copyright holder.
To view a copy of this license, visit https://creativecommons.org/licenses/by/4.0/

Point-by-point Response to Reviewer Comments

we want to thank the editor and the reviewers for the opportunity to revise and resubmit our manuscript, now titled "Multi-omics Integration Unravels Four Molecular Subgroups of Corticotroph Pituitary Neuroendocrine Tumours with Distinct Clinicopathological Features". We appreciate the time and effort you have dedicated to evaluating our work.
We have carefully considered the reviewers' comments and have thoroughly modified the manuscript with major revisions. We believe these comments were valuable and have fundamentally contributed to improving the clarity and quality of the article. Below, we provide a detailed point-by-point response to all comments. Our responses here and all corresponding changes in the manuscript have been coloured in blue.

Reviewer Comments

Reviewer #1, expertise in multi-omics for pituitary neuroendocrine tumours (Remarks to the Author):

The authors present a multi-omic analysis of 270 corticotroph PitNETs, integrating epigenomic, transcriptomic, proteomic, and histopathologic data. In their analysis, the authors identify 4 distinct molecular subgroups of corticotroph PitNETs. Subgroup 1 contained mixed clinical presentation of both silent and overt Cushing disease, but was defined by numerous copy number alterations compared to the other subgroups. Subgroups 2 and 3 are mainly comprised of clinically silent corticotroph PitNETs and were distinguished by SSTR1 expression in subgroup 2 and GATA3 expression in subgroup 3. Subgroup 4 comprised of mainly overt Cushing disease presentation, along with increased expression of SSTR5. Additionally, the authors developed a methylation classifier for others to stratify specimens into their reported schema.
Based on the evidence as presented, the presence of 4 molecular groups is convincing, but the clinical significance of these groups is difficult to ascertain, as acknowledged by the authors. In part this is due to the clinical metadata associated with these samples, which is of course important when associating subgroups with clinical significance.

Major comments:
1. Can the authors clarify what clinical factors were used to determine "overt Cushing"? In the 6 cases of "subclinical Cushing" what led to the discovery of biochemical hypercortisolism in these patients? How was hypercortisolism determined in both "subclinical Cushing" and "overt Cushing" patients?

We thank the reviewer for this important comment. We have revised the Methods section to clarify the definitions of "subclinical" and "overt" Cushing's disease and to specify how hypercortisolism was assessed. Testing for hypercortisolism was performed as part of the routine endocrinological evaluation of patients with pituitary lesions. This comprised low-dose dexamethasone suppression testing, assessment of urinary free cortisol and late-night salivary cortisol. Pathological measurements in either or multiple of these tests defined hypercortisolism biochemically.
Subclinical CD was defined as biochemical evidence of hypercortisolism in the absence of overt clinical features of Cushing's syndrome, including, e.g., central weight gain, facial plethora, proximal myopathy, hypertension, diabetes mellitus, and/or skin changes such as easy bruising or striae, as assessed during routine clinical evaluation.

Overt CD was defined by the presence of both biochemical evidence of hypercortisolism and clinical features compatible with Cushing's syndrome. We have added this information to the Methods section (p.14, lines 11-20).

2. In Supplementary File 1, it seems that some of the tumors used in the series are from reoperation cases or metastasis resections. These tumors should be excluded or analyzed separately as comparing metastases or tumors that have undergone adjuvant therapy/experienced recurrence may introducing confounding genomic profiles when comparing to primary tumors.

Thank you for raising this important point. We agree that prior surgical or medical interventions could potentially influence global molecular profiles and thus represent a source of confounding factors.
To address this concern, we performed global epigenomic clustering analyses including all tumors of the series and specifically assessed whether any samples would show tendencies to segregate according to surgical settings (primary vs. reoperation or metastasectomy) or prior radio-/chemotherapy. We did not observe any discernible clustering effects in relation to these factors within or across the molecular subgroups, indicating that prior interventions did not evidently impact the global epigenomic tumor profiles in our dataset.
As this point provides valuable insight, we have added these results to the manuscript in the new Supplementary Figure 4.

3. Outcomes regarding surgical resection should be reported if available, especially for cases involving cavernous sinus involvement or tumors that are adjacent to the cavernous sinus. In these cases, was the medial wall of the cavernous sinus resected? This is important to specify as remission risk can be reduced by medial cavernous sinus wall resection in appropriate cases

We thank the reviewer for this important and relevant comment. In response, we revisited the available clinical documentation and expanded our dataset to include the extent of surgical resection (gross total, subtotal, partial), which was available for a total of 89 cases. These data have now been added to the revised Supplementary Data 1 and incorporated into our integrated stratification model for prognostication (see also upcoming comment #5).
Medial wall resection of the cavernous sinus is performed at our institution in selected cases where involvement is suspected. However, this specific surgical detail was not consistently or systematically documented in operative reports and therefore could not be reliably extracted for the series in a retrospective manner. We acknowledge the relevance of surgical techniques for postsurgical outcomes and have included this into the Discussion (p.9, line 51 - p.10, line 3).

4. The authors' clinical data is limited based on lack of follow up. There are many patients within their own series that do not have any follow-up documented, yet have clinical data completed (i.e. Patient 25 and 26). However, how can the authors know if the patients truly do not have any recurrence, adjuvant therapies, metastasis, etc. if there is no follow up to base these conclusions on? Additionally, there are only 32 patients that have >15 months follow up. Given that recurrence in Cushing disease commonly occurs > 15 months following initial intervention, the current follow-up definition of 6 months as "single-surgery controlled" is not sufficient to accounts for the majority of cases that recur at longer follow up. This also affects the authors ability to characterize tumors as "high", "moderate", and "low" risk effectively since this outcome is based on whether tumors recur at a 6 month interval. Is there an opportunity for the authors to supplement the existing data with data for patients from their own institution at least?

We thank the reviewer for highlighting these important concerns. We agree that limited follow-up constrains the assessment of recurrence, long-term disease control, and required therapeutic interventions. Given the retrospective nature of the series and the integration of external datasets, detailed time-to-event analyses (e.g., Kaplan–Meier estimates) were unfortunately not ascertainable in a consistent and reliable manner across all cases. We therefore adopted an ordinal outcome framework to group patients by overall clinical trajectory while avoiding overinterpretation of incomplete longitudinal data.
To address the reviewer's concerns, we have made the following revisions:
1. Refinement of outcome definitions: We agree that a 6-month follow-up is insufficient to define durable disease control. Accordingly, we have renamed the category "single-surgery-controlled" to "initial remission" to more accurately reflect the limited follow-up window.
2. Performance analysis with extended follow-up: We have refined our integrated clinicopathological stratification model (new Figure 7 and new Supplementary Figure 6a, see also upcoming comment #5) and tested the performances of all investigated stratification models when progressively restricting the series to cases with longer minimum follow-up durations. We now show a consistent superior performance of our proposed model compared to other existing stratification approaches (new Supplementary Figure 6b-d).
3. Handling of missing follow-up: For patients without documented follow-up (e.g. cases 25 and 26), clinical trajectories are no longer inferred in the revised Supplementary Data 1 (these variables initially referred to the status at time of surgery for these cases). The corresponding elements are now reclassified as "NA" to follow data consistency and avoid misinterpretation of the provided data.
4. Data update: We have updated clinical follow-up data wherever possible within our institutional case series, and these revisions have been incorporated into Supplementary Data 1 and subsequent analyses. We were able to update follow-up data for a total of 13 cases.
5. Further clarification of limitations: We have expanded the Discussion to explicitly acknowledge the limitations imposed by incomplete follow-up and the need for prospective studies to validate the proposed risk stratification model (p.9, line 46 – p.10, line 3).
Thank you again, we believe these revisions have tremendously improved the transparency and robustness of our analyses.

5. When determining risk stratification, the authors investigated the predictive value of WHO classification, Trouillas classification, and molecular subgroups. However, the authors could additionally investigate a univariate analysis followed by a multivariate analysis of all clinical and molecular features to determine which variables are predictive of disease outcomes in order to capture other variables such as gender, age, preoperative ACTH/cortisol levels, etc.

This is an insightful and constructive suggestion, which has led us to substantially refine and improve our integrated stratification model for prognostication.
In response, we performed both univariable and multivariable ordinal regression analyses incorporating all available clinicopathological and molecular variables (including surgery extension, see earlier comment #1). In both univariable and multivariable analyses, gross total resection, invasion, and molecular subgroup 1 remained independent significant predictors of clinical outcome, whereas other variables did not show consistent independent associations. These findings were further supported by the updated recursive partitioning analysis, in which these same three variables prevailed as key determinants of clinical trajectories.
The results of these analyses have been incorporated into the revised manuscript (Figure 7 and Supplementary Figure 6), and the Results and Methods sections have been updated accordingly (p.8 lines 10-28 and p.14 lines 41-51).

6. On Page 19, "Incorporation of corticotroph molecular subgroups into currently established

prognostication frameworks": how many samples were used to generate this prognostic data and classification tree? Ideally, only samples that have the full complement of data (i.e. no missing data in Supplementary File 1) should be included for this analysis. Please indicate in Supplementary File 1 which samples were used to create the classification decision tree.

We thank the reviewer for this helpful comment. The prognostic model and classification tree were indeed constructed incorporating only samples that met all predefined criteria without missing values. Exceptions were made only for cases already assigned to the highest-risk category, which was based on the occurrence of defining events and did not require additional follow-up data once these events had occurred. In total, 106 cases were incorporated. Of these, 80 cases were of the reference series (47 of which were internally derived) and 26 cases were of the validation series (all of which were internally derived). To ensure transparency, Supplementary Data 1 has been revised to explicitly display the clinical trajectories of all cases. Accordingly, samples not included in the models are marked as "NA" and the number of cases used has been disclosed in the legend of Figure 7.
In addition, we have expanded the titles and legends of all figures, where appropriate, to clarify the sample selection for each analysis (see also response to comment #2 of Reviewer #2).

Minor comments:
Page 11, Line 2: How was it decided which samples would be included in the reference series versus the validation series?

The allocation of samples to the reference and validation series was based on the chronological availability of samples, with earlier cases assigned to the reference series and subsequently collected cases assigned to the validation series. For clarity and transparency, we have added a new subsection entitled "Study design and series assignment" to the Methods section, where this process is now described in further detail (p.13 lines 11-16).

Page 20, Lines 17-18: Should this be 98.5% (not 0.985%)?

We thank the reviewer for catching this error, which has been corrected in the revised manuscript (p.8 line 47).

Reviewer #2, expertise in corticotroph pituitary neuroendocrine tumour classification (Remarks to the Author):

Dear Authors,

I have read your submitted manuscript entitled "Integrated Multi-omics Analyses Classify Corticotroph Pituitary Neuroendocrine Tumors into Four Molecular Subgroups with Distinct Clinicopathological Features" carefully and I have to make the following comments:

1. The idea of finding any differences at the molecular level among the corticotroph PitNETs is interesting, in particular with respect to the variable biological behavior of this group of PitNETs. However, I would like to ask you to elaborate more on the advantages of your proposed categorization in these four molecular subgroups and discuss about its superiority compared to the existing subgrouping in the current WHO Classification. You conclude in the results section "In summary, histomorphological features mainly differentiated subgroups 1 and 4 from subgroups 2 and 3, yet they did not reliably distinguish among all four molecular subgroups." Why should your proposed subgrouping be favoured over the existing classification?

We thank the reviewer for this important remark. We agree that the proposed molecular classification required further evaluation in relation to the current WHO classification framework. Therefore, we have implemented additional analyses in the revised manuscript which further support the potential value of the proposed molecular-based subgrouping.
Specifically, multivariable ordinal regression analyses identified molecular subgroup 1 as an independent negative prognostic factor, surpassing the prognostic impact of other pathological features (Figure 7, see also response to comment #5 of reviewer #1).
Additionally, we demonstrate that the identification of the proposed molecular subgroups using surrogate marker immunohistochemistry showed improved reproducibility compared to the current standards of histological subtyping, with reduced interobserver variability in subgroup assignment (Figures 3+4, see also upcoming comment #3).
Of note, we have removed the mentioned sentence from the Results section to avoid premature interpretation, as we agree that its contextual implications are more appropriately addressed in the Discussion (p.9 lines 31-38; p.10 lines 4-7).

2. You describe various experimental procedures that you carried out but in the way that they are presented they may confuse the reader. For example, you use at the beginning for the initial experiments a number of cases then you add several other numbers of cases (either internal or external) and at the end one tries to identify what belongs to what. Therefore, I would suggest amending your methods section to be better organized, thus avoiding any perplexity.

We thank the reviewer for this suggestion. In response, we have substantially revised the Methods section to improve clarity and structure. Specifically, we now first describe the internal series and all investigations directly related to it. This is followed by a dedicated section on the external series and its integration into the internal series. Subsequently, we clarify sample assignment to the reference and validation series. All methods applicable to both series are presented in the then following methods sections.
In addition, we have revised all relevant figure legends to indicate which samples were included in which analysis.
We find these changes have improved the traceability of sample usage throughout the manuscript.

3. As far as immunostaining assessment and histological subtyping are concerned, the method that you described has a high degree of subjectivity; it depends on the "observer's eye". I do not think that it is possible for the neuro-(pathologist) to recognize small differences, like 66% and 67%. However, this is of paramount significance, as in the first case the tumor will be considered of intermediate positivity whereas in the second of abundant positivity for the corresponding marker. In order to solve this issue, I would suggest to use a more objective approach, for example through the use of an automated counting apparatus. Another option would be to examine interobserver variability and make the appropriate analytical adjustments.

We thank the reviewer for this important and insightful comment. We fully agree that immunohistochemical scoring and histological subtyping may be subject to interobserver variability. As automated image analysis is often not yet implemented in routine diagnostic (neuro)pathology, and to ensure applicability in real-world practice, we addressed this concern by revising our histopathological scoring strategy to incorporate interobserver assessment.
All cases were independently evaluated by two board-certified neuropathologists, which individually scored stainings for markers relating to the WHO subtypes and the molecular subgroups. Mean scorings were used as a basis for the histopathological subtyping of all cases. This revised approach allowed us to also specifically assess interobserver agreement and disagreement for all relevant stainings. We found that scoring of surrogate marker stainings was indeed associated with substantially higher interobserver agreement compared to WHO histological subtyping (Figure 3+4, Supplementary Data 7). Notably, based on this new approach, we were able to refine our cutoff to a more intuitive value of >50% positive tumor cells to determine abundant surrogate marker staining with high specificity in our series. The revised Figure 4 also shows that tumors with immunopositivity near this cut-off were indeed quite rare and that the assignment of most cases was unequivocal.

4. I would like to kindly ask you to explain the controversial results, on the one hand the Ki-67 (a well established and widely used proliferation marker) is more abundant in group 4 tumors, which on the other hand these are the ones that have the most favourable prognosis. We must consider that this marker is used as integral part of the grading of some tumors, in particular the neuroendocrine neoplasms; the higher the Ki-67 expression the higher the grade of the tumor.

We thank the reviewer for this insightful comment. We agree that the relationship between the molecular subgroups and Ki-67 appears counter-intuitive given the generally established role of proliferative activity as a grading parameter in neuroendocrine neoplasms.
While speculative, our findings suggest that in corticotroph PitNETs Ki-67 may not serve as a reliable prognostic marker in the same manner as it does in other neuroendocrine neoplasms. On the other hand, the commonly used 3% cutoff may need revision in pituitary tumors. We have added these discussion points to the revised manuscript (p.9, lines 40-45).
Of note, our revised immunostaining assessment approach incorporating two independent observers did not alter the finding of high proliferative activity in subgroup 4. However, the results of the revised prognostic stratification analyses show that adverse outcomes primarily associated with molecular subgroup 1, whereas subgroup 4 alone did not demonstrate a statistically significant positive prognostic effect (Figure 7).

5. Regarding the included images, acquired from the performed histochemical and immunohistochemical stains, these should be improved and be replaced with others of higher magnifications, where one can clearly identify the various staining patterns (i.e., membranous, cytoplasmic and nuclear).

We thank the reviewer for this helpful suggestion. We have replaced the previously shown histological and immunohistochemical images in Figures 3 and 4 as well as in Supplementary Figure 5 with representative fields in higher magnification. Scale bar descriptions in the legends have been adjusted accordingly. All figures are provided as high-resolution png files in the revised version of the manuscript.

6. Further, in the Figure 4.: B-E should be replaced by C-F, G-J by I-L, L-O by O-R. Similarly the G and H and the L and M do not correspond well to what it is written in the relevant part of the figure legend.

We appreciate the reviewer carefully reading the manuscript. These were indeed errors in the figure legend. The legends have been corrected accordingly.

Furthermore, in Figure 5 there are different numbers of cohorts (e.g., patient sex: 270 cases, clinical presentation: 236 cases, tumor size: 244 cases). I do not really understand why there are these differences in the cohorts, and I could not find the explanation of these differences anywhere within the text.

Thank you for this important comment. The differences in numbers across the subpanels in Figure 5 arise from incomplete clinicopathological annotations in both internal and external cases. To address this, we have included a sunburst plot in Figure 5a for visualization of data completeness and clarified data origins in the respective legend and also in the legends of other figures to ensure transparency across the manuscript. Additionally, the new Supplementary Data 2 has been included to display the origin and molecular reclassification of all external samples for full reproducibility of our findings.

Thank you in advance for considering my suggestions, which I hope will contribute to the improvement of the manuscript.

Kind regards,
The Reviewer

We hope that the revisions have adequately addressed the reviewers' comments and that the manuscript is now suitable for publication. We sincerely appreciate your consideration and look forward to your feedback.

Sincerely,
Matthias Dottermusch

Response to Reviewer Comments

We want to thank the editor and the reviewers for the time and effort they have dedicated to evaluating our manuscript, titled "Multi-omics Integration Unravels Four Molecular Subgroups of Corticotroph Pituitary Neuroendocrine Tumours with Distinct Clinicopathological Features". The reviewers' final comments are shown below.

Reviewer Comments

Reviewer #1 (Remarks to the Author):

The authors have now revised their manuscript and addressed my criticisms satisfactorily. Ultimately, although still with limitations regarding clinical significance, the manuscript is of interest. Notably, of the 4 molecular groups identified, only one is of apparent clinical significance (molecular group 1). The remaining groups may later be discovered to be of some importance, as well, but this manuscript provides, at minimum, basis to further investigate group 1 tumors in future studies.

Reviewer #2 (Remarks to the Author):
Dear Authors,

Thank you for amending and improving your manuscript according to my comments.
I am satisfied of the final version of your paper and I recommend its acceptance and publication on the journal "Nature Communications".

Kind regards,

The Reviewer

We thank the reviewers for their positive evaluation of our work. No further changes have been made to the contents of the article. As requested, only editorial revisions have been made, and we hope that the manuscript is now suitable for publication in Nature Communications.

Sincerely,
Matthias Dottermusch
