# Supplementary material for: Multi-omics integration unravels four molecular subgroups of corticotroph pituitary neuroendocrine tumours with distinct clinicopathological features
Source: Nat Commun. 2026 Aug 4;17:7777. doi: 10.1038/s41467-026-76292-y (PMC13438112; doi:10.1038/s41467-026-76292-y)
Supplement: Supplementary file 3 [file 41467_2026_76292_MOESM1_ESM.pdf]

Multi-omics Integration Unravels Four Molecular Subgroups of Corticotroph Pituitary Neuroendocrine Tumours with Distinct Clinicopathological Features

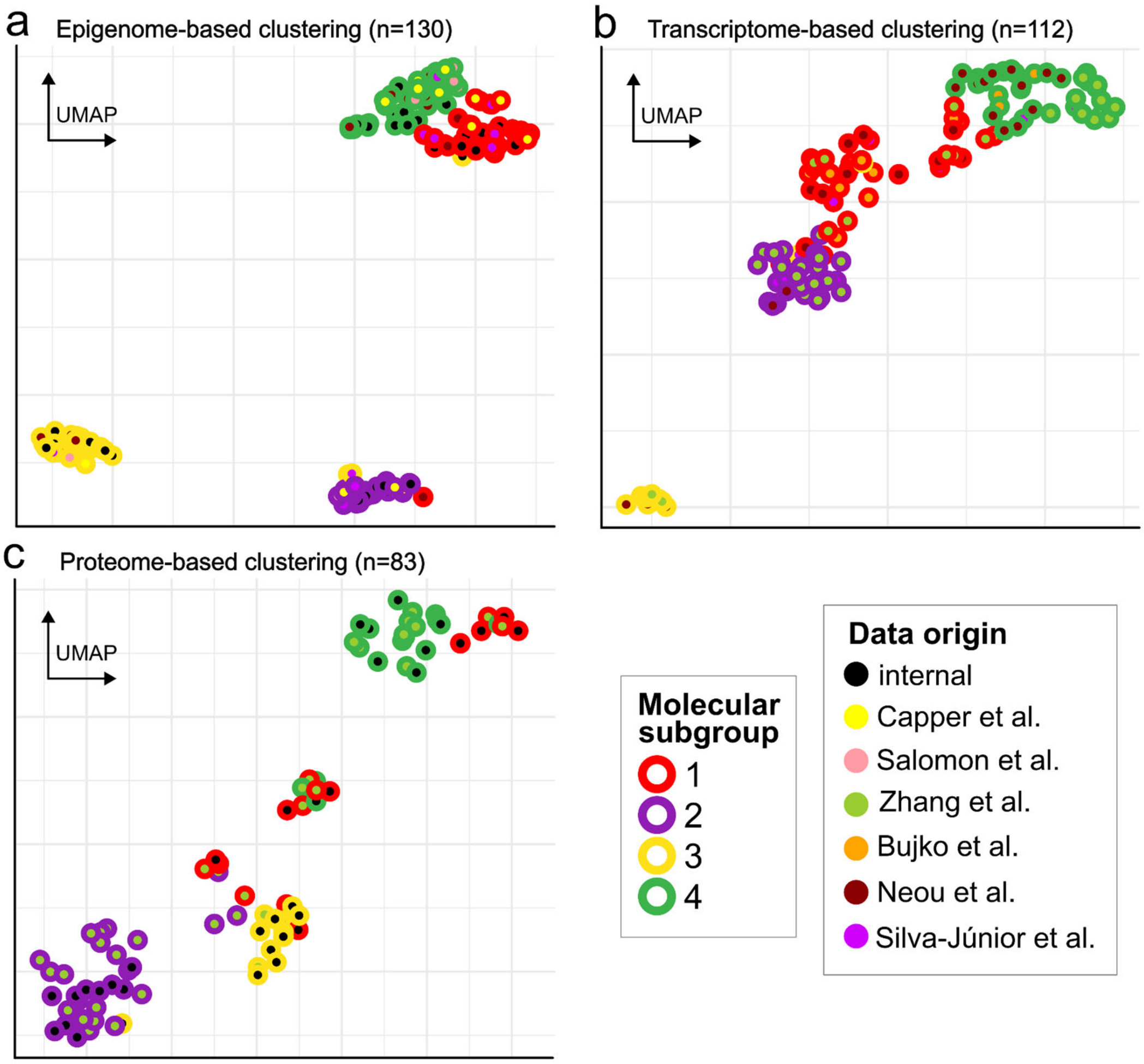

Supplementary Figure 1: UMAP based molecular clustering of corticotroph PitNETs across studies (related to Figure 1)

UMAP clustering recapitulated four molecular subgroups in corticotroph PitNETs based on the top 2500 variant DNA methylation beta-values (a), based on the top 250 variant genes (b), and based on the top 250 variant proteins (c). Circle borders conform to the molecular subgroups as assigned in Figure 1i.

Sample sizes (n) indicate the number of tumour samples. All analyses shown were based on reference series samples, for which corresponding data were available [1–6]. Source data are provided as a Source Data file.

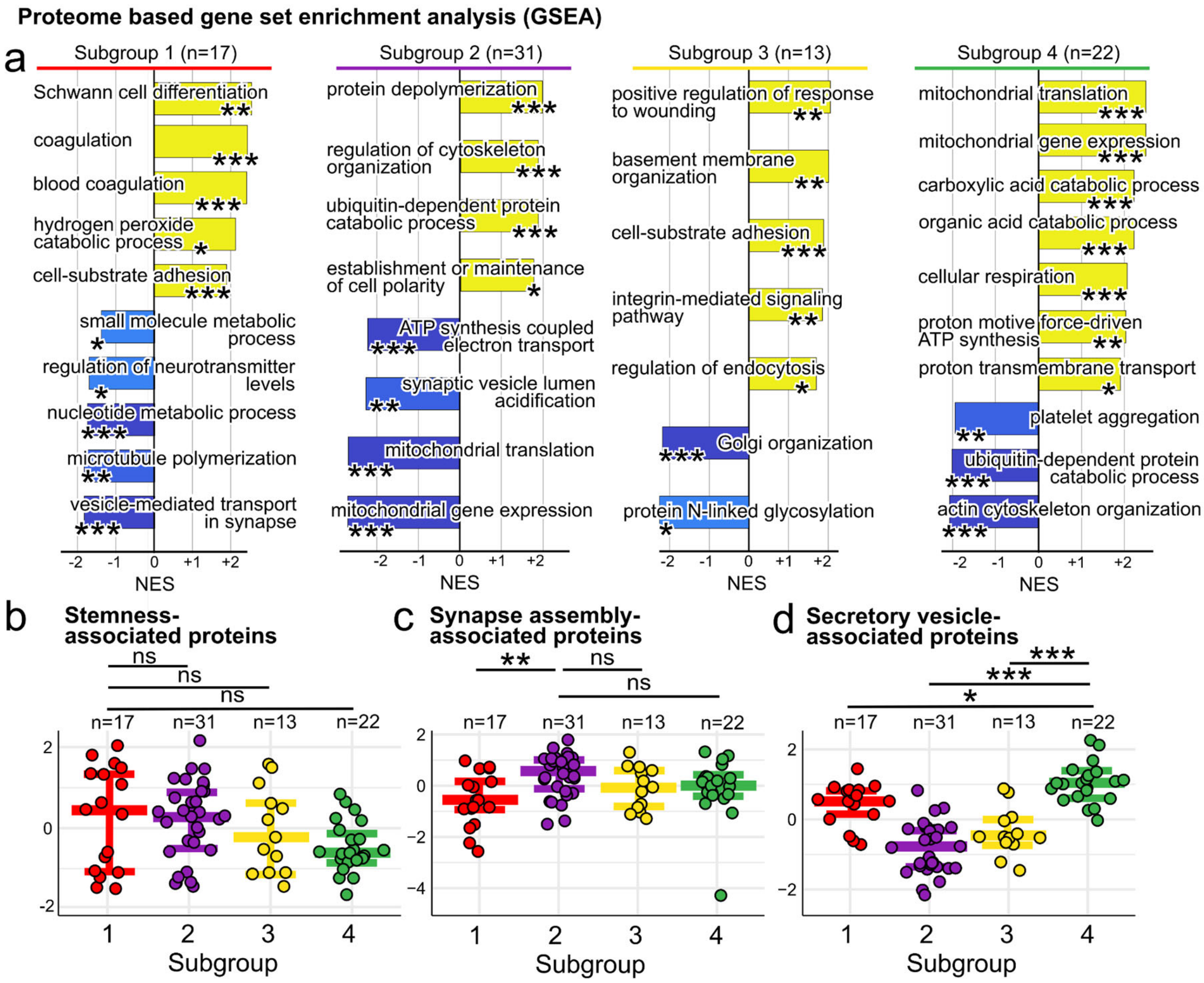

Supplementary Figure 2: Overview of proteome-based features of corticotroph molecular subgroups (related to Figure 2)

a Proteome based gene set enrichment analyses (GSEA) across the four subgroups revealed specific protein expression profiles. The maximum top 10 most significantly enriched gene sets of each subgroup are shown. Permutation-based gene set enrichment analysis. Gene ontology terms were reduced to remove semantic redundancy. Abbreviation: NES = normalized enrichment score.

b-d Proteome based single-sample GSEA revealed enrichment of the top 5 variant proteins of the gene sets “stemness”, “synapse assembly”, and “secretory vesicle”, as represented in the proteome data. No members of the “gonadotrophs” gene set were found in the proteome data. Horizontal bars indicate the interquartile range (IQR, 25th-75th percentiles), with central lines marking medians. Two-sided Student’s t-test

Sample sizes (n) indicate the number of tumour samples. p-values were adjusted for multiple comparisons using Bonferroni correction: not significant (ns) p≥0.05, *p<0.05, **p<0.01, ***p<0.001. All analyses shown were based on reference series samples, for which proteome data was available. Source data are provided as a Source Data file.

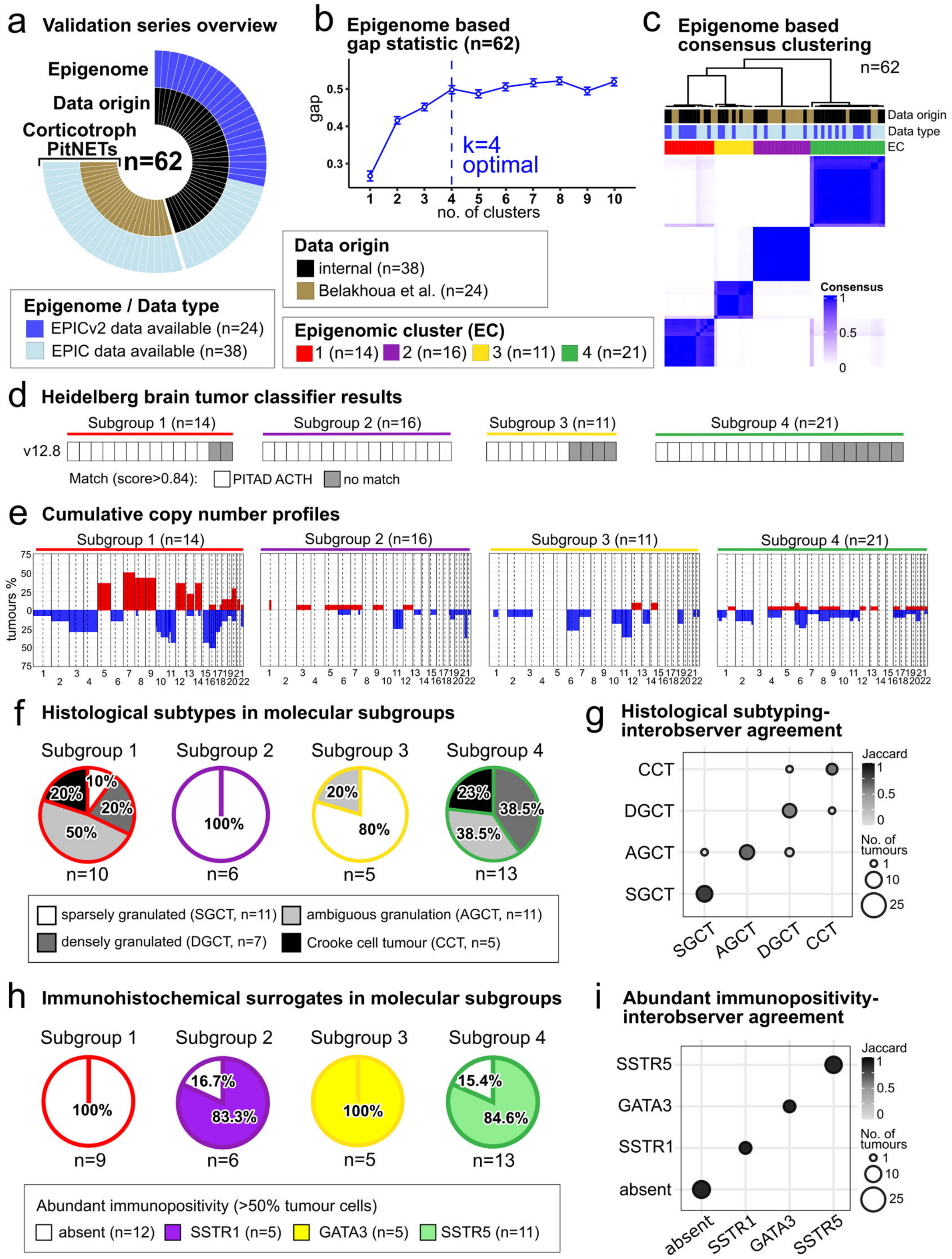

Supplementary Figure 3: Molecular and histomorphological features of corticotroph molecular subgroups are confirmed in an independent validation series (related to Figures 1-4)

a Sunburst chart shows an overview of the validation series, which was investigated for epigenomic signatures. Available DNA methylation data array types and respective data origins are shown for each sample.

b Gap statistics demonstrated the optimal number of clusters k to be 4 based on epigenome data. Data points show the estimated gap statistic, error bars indicate ±1 Monte Carlo standard errors.

c Consensus clustering in conjunction with epigenome data of the reference series showed the distribution of samples among the four molecular clusters. Batch effects relating to different data origins or array types were not apparent. Clustering was based on the top 2500 variant DNA methylation beta-values.

d The Heidelberg brain tumor methylation classifier v12.8 confirmed corticotroph PitNET diagnosis in most cases across the four molecular subgroups 1-4.

e Cumulative copy number profiles of the molecular subgroups 1-4 are shown. As demonstrated in the reference series, subgroup 1 tumours harboured the most frequent and abundant copy number alterations.

f Pie graphs show the proportions of histological subtypes among the four molecular subgroups for internal cases, providing a similar distribution as shown in the reference series.
g Agreement in histological subtyping of two independent observers is shown for all internal cases. Cohen's kappa=0.801, p=1.55e-15, n=34.
h Mutually exclusive, abundant immunopositivity for either SSTR1, GATA3 or SSTR5 in internal cases was highly specific for the subgroups 2, 3 and 4, respectively.
i Agreement in assessing abundant immunopositivity for SSTR1, GATA3 and SSTR5 of two independent observers is shown for all internal cases. Cohen's kappa=1, p=1.5e-23, n=33.
Sample size (n) indicates the number of tumour samples. All analyses shown were based on validation series samples, for which corresponding data were available [7]. Source data are provided as a Source Data file.

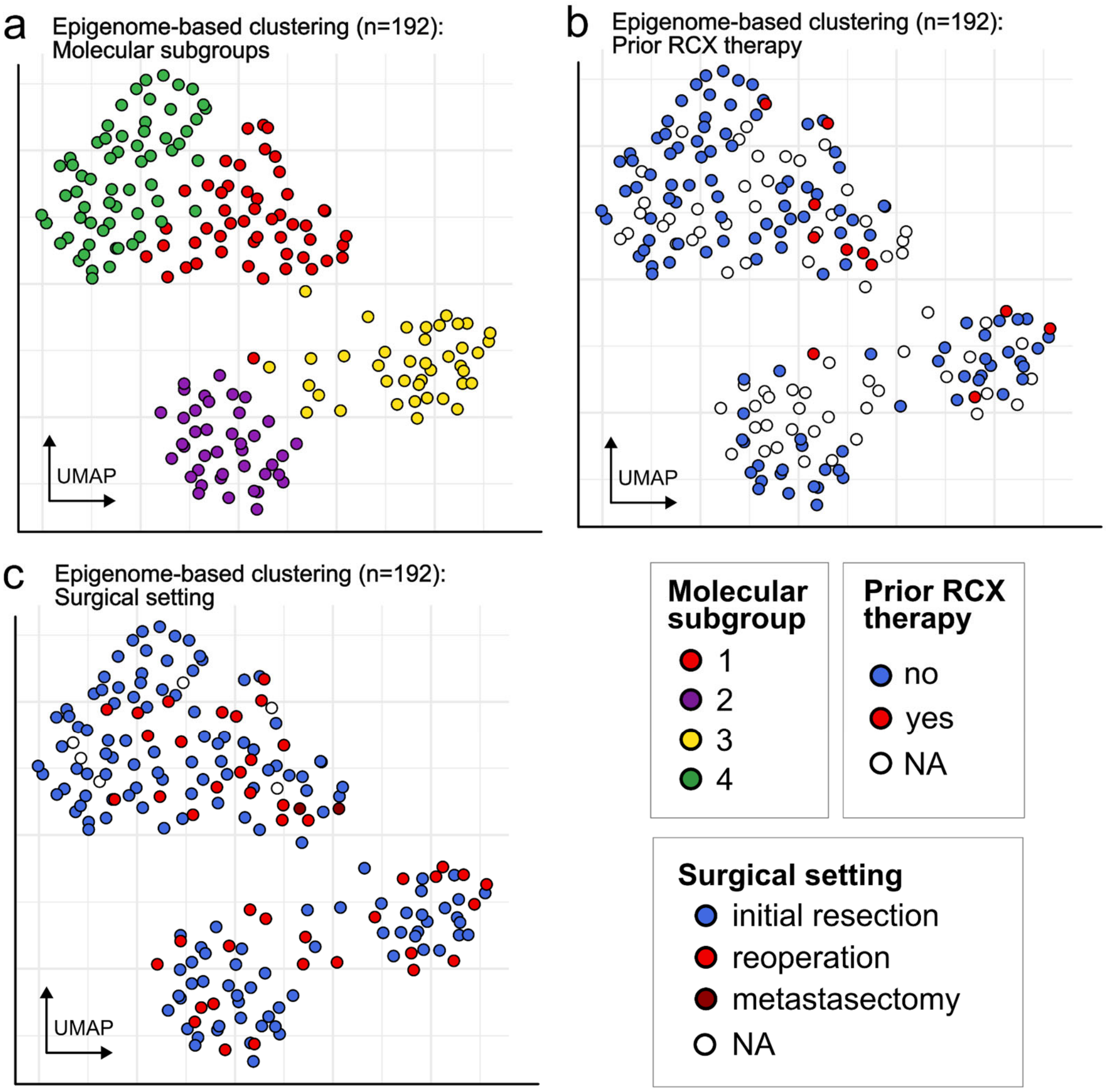

Supplementary Figure 4: Epigenomic clusters are not impacted by prior treatments in corticotroph PitNETs (related to Figure 1 and Supplementary Figure 3)

UMAP clustering robustly recapitulated the four molecular subgroups in all corticotroph PitNETs of the reference and the validation series combined (a). Prior tumour treatment with radio-/chemotherapy (RCX, b) or surgical setting (c) did not evidently impact global epigenomic profiles.

Clustering was based on the top 2500 variant DNA methylation beta-values. Sample sizes (n) indicate the number of tumour samples. Source data are provided as a Source Data file.

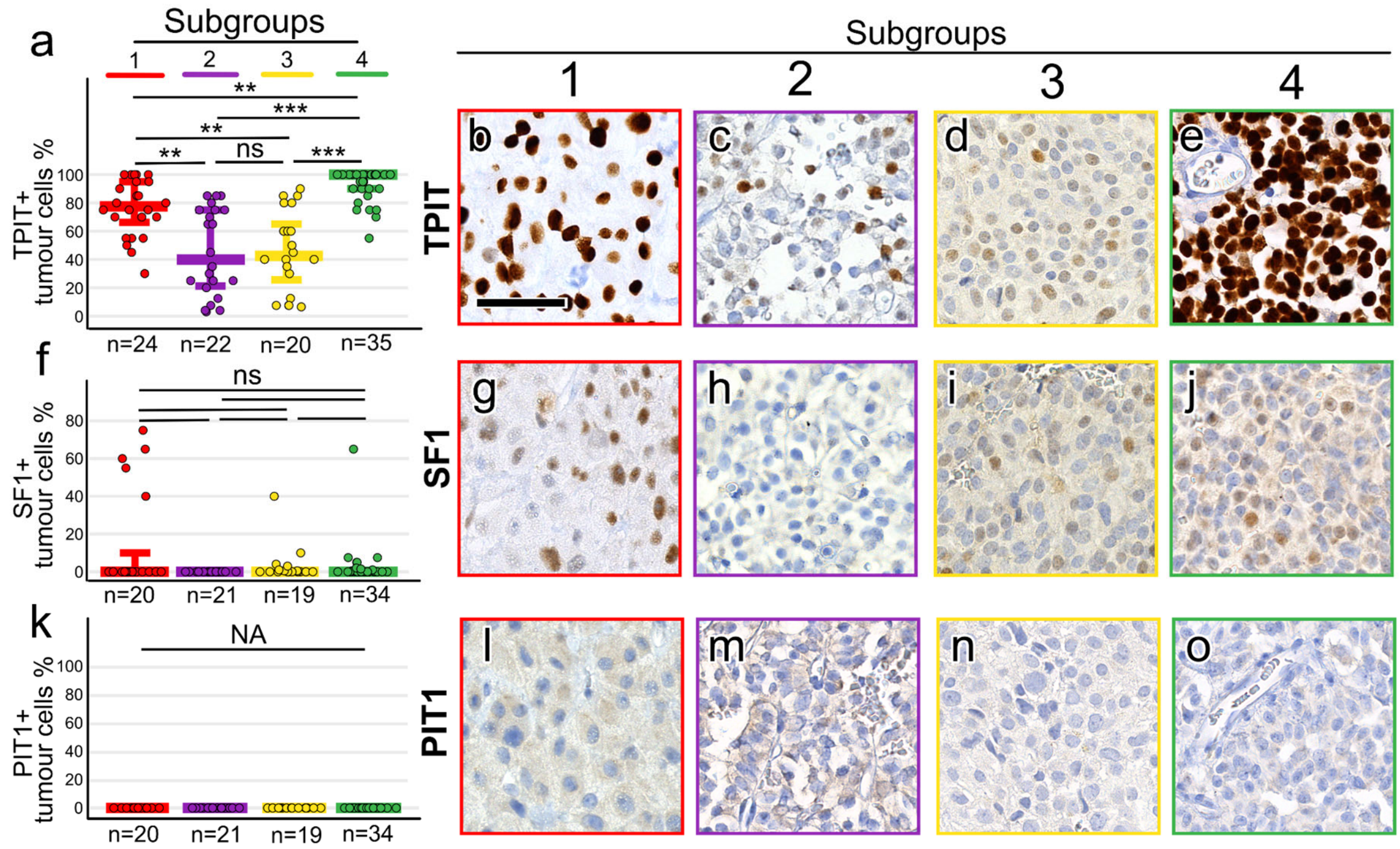

Supplementary Figure 5: Transcription factor expression in corticotroph PitNET molecular subgroups (related to Figures 3-5).

a-e Abundant nuclear TPIT staining was mainly found in subgroups 1 and 4. Representative histopathological images of subgroup 1 (n=24), 2 (n=22), 3 (n=20), and 4 (n=35) tumours are shown in b-e, respectively.

f-j The majority of corticotroph PitNETs were immunonegative for SF1. Partial SF1 immunopositivity was encountered in a subset of cases with no evident differences between the molecular subgroups 1-4. Representative histopathological images of SF1 immunopositive tumours of subgroup 1 (n=5), 3 (n=7), and 4 (n=9) are shown in g, i and j, respectively. A representative histopathological image of a SF1 immunonegative subgroup 2 tumour (n=21) is shown in h.

k-o No tumours with nuclear PIT1 immunopositivity were encountered. Representative histopathological images of subgroup 1 (n=20), 2 (n=21), 3 (n=19), and 4 (n=34) tumours are shown in l-o, respectively.

Sample sizes (n) indicate the number of tumour samples. Horizontal bars indicate the interquartile range (IQR, 25th–75th percentiles), with central lines marking medians. Percentages based on the mean quantifications of two independent observers are shown. For all shown statistical analyses p-values were calculated using two-sided Student's t-test and adjusted for multiple comparisons using Bonferroni correction: not significant (ns) $p \geq 0.05$, *$p<0.05$, **$p<0.01$, ***$p<0.001$, NA = not available (mathematically non-computable). Scale bar is 50µm in all histological images. All analyses shown were based on internal reference and validation series samples, for which corresponding data were available. Source data are provided as a Source Data file.

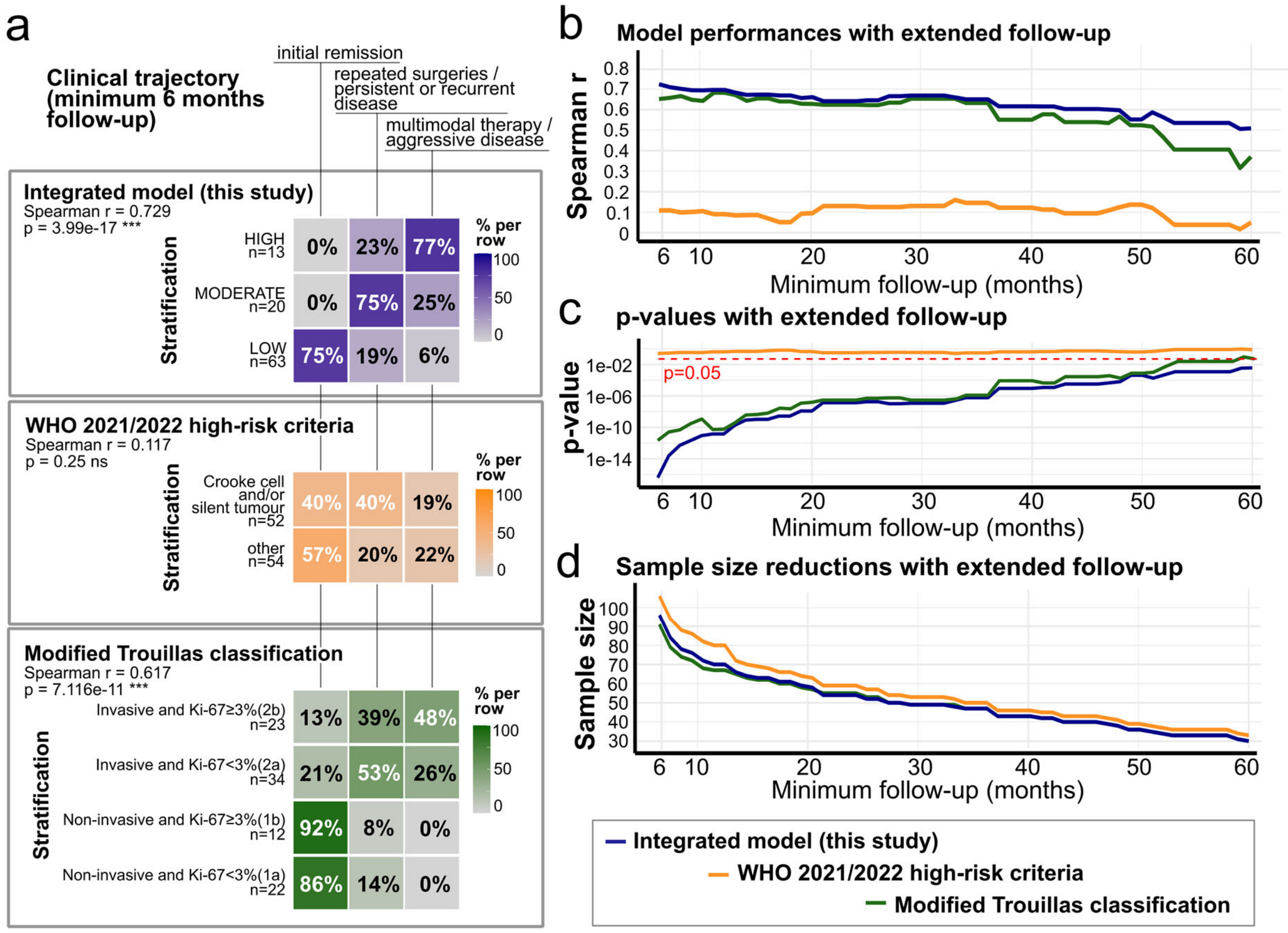

Supplementary Figure 6: Comparison of different stratification models (related to Figure 7)

a Stratification of cases based on the integrated model, WHO high-risk criteria [8] and modified principles of the five-tiered classification of Trouillas *et al.* [9] are shown. Considering a minimum follow-up time of 6 months (as used to construct the tree shown in Figure 7c), the integrated model demonstrated improved risk stratification compared to the other two approaches. Of note: Trouillas' five-tier classification includes Ki-67 index, mitotic counts, and p53 immunostaining in the definition of proliferation [9]; in our analysis, mitotic counts and p53 staining were not consistently reported, and proliferation was only determined by Ki-67 index cutoff ≥3%.

b-d Increasing the minimum required follow-up time within the case series preserves the superior performance of the integrated model, as reflected by Spearman's r (b) and corresponding p-values (c). Sample sizes remained comparable across the three approaches (d).

Sample sizes (n) indicate the number of cases. All analyses shown were based on reference and validation series samples, for which sufficient follow-up data were available. Source Data are provided as a Source Data file.

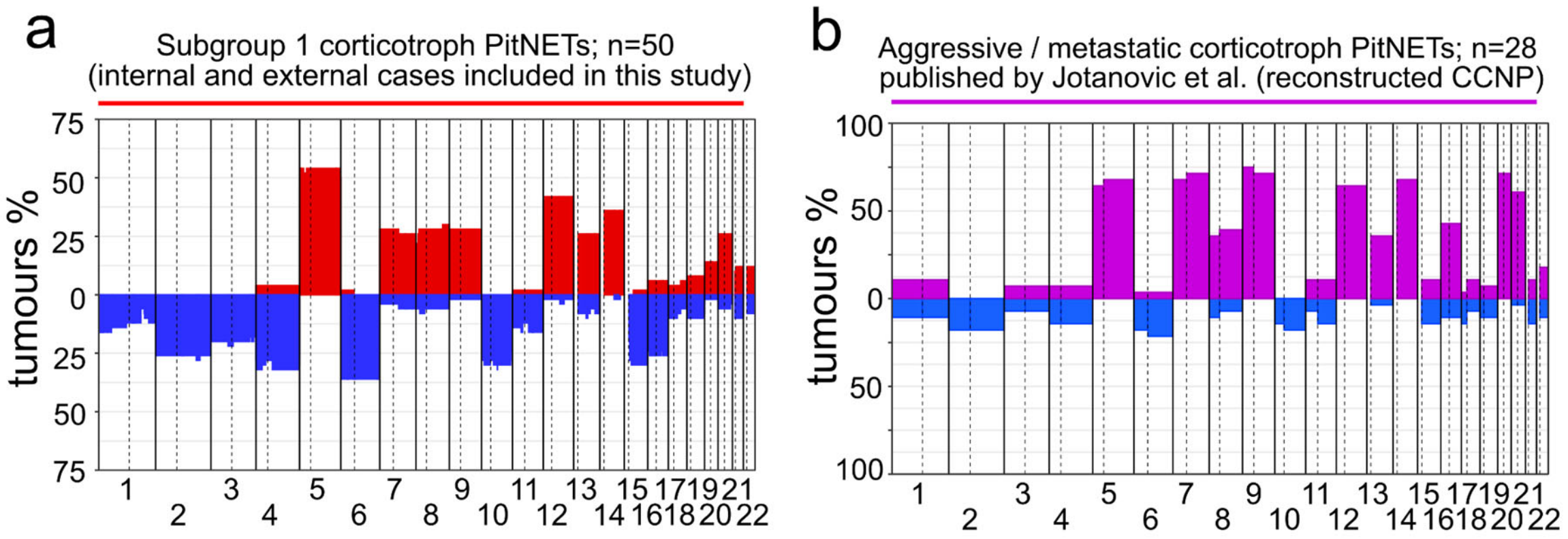

Supplementary Figure 7: Chromosomal alterations in subgroup 1 tumours correspond to those of external aggressive and metastatic corticotroph PitNETs (related to Figures 2 and 7)

a Cumulative copy number profile (CCNP) is shown for all 50 subgroup 1 tumours of this study (internal and external cases of the reference and validation series), for which corresponding data were available. Gains in chromosomes 5, 7-9, and 12, 13q, 14q and 20 are among the most prominent patterns.

b CCNP is shown for an external independent case series previously published in 2025 by Jotanovic *et al.* [10], which included 28 aggressive and/or metastatic corticotroph PitNETs. The shown plot was constructed based on the figures of this external independent publication. Chromosomal alteration patterns indicate that this external case series was enriched with subgroup 1 tumours.

Sample sizes (n) indicate the number of tumour samples. Source data are provided as a Source Data file.

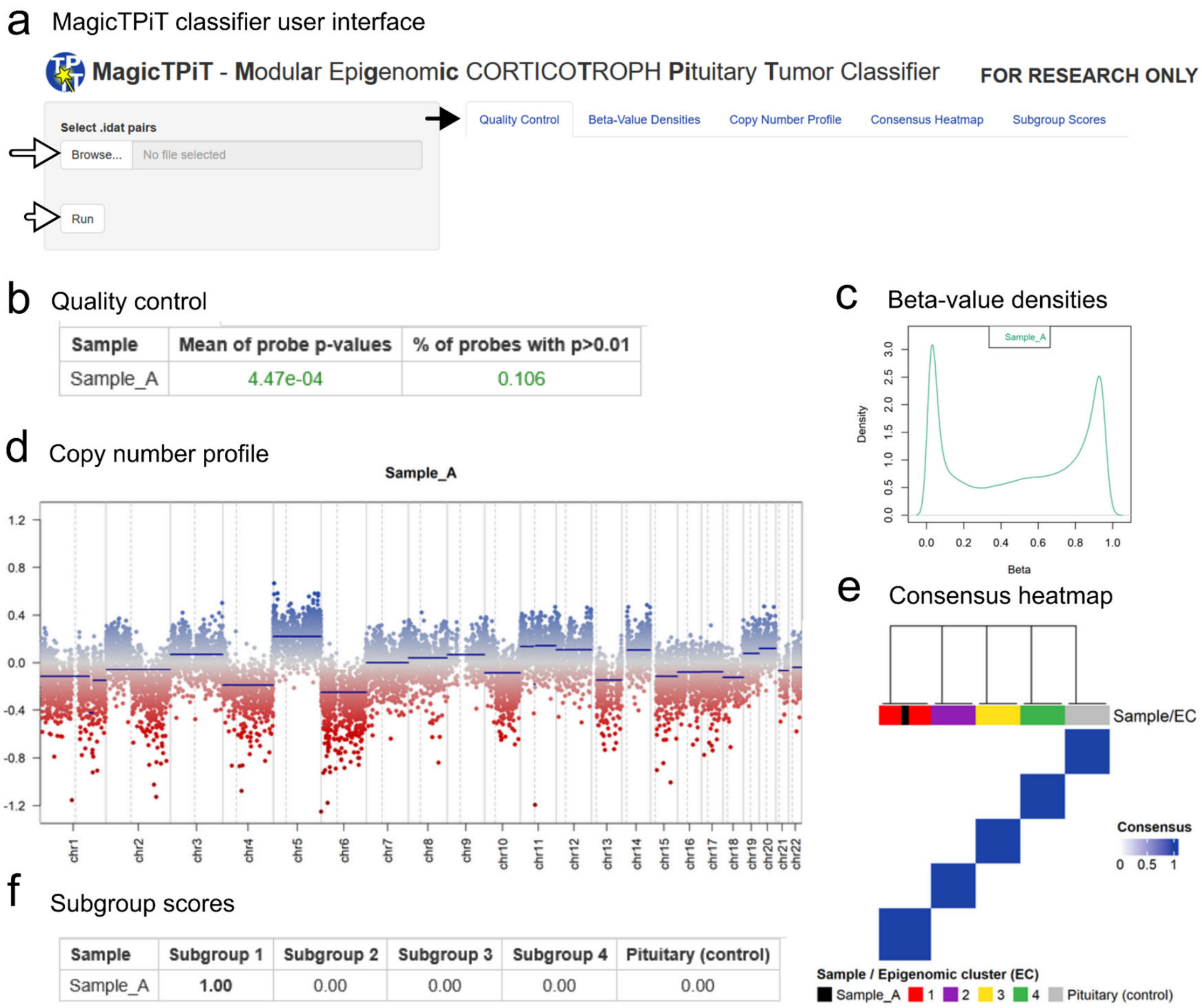

Sample	Mean of probe p-values	% of probes with p>0.01
Sample_A	4.47e-04	0.106

Sample	Subgroup 1	Subgroup 2	Subgroup 3	Subgroup 4	Pituitary (control)
Sample_A	1.00	0.00	0.00	0.00	0.00

Supplementary Figure 8: MagicTPiT classifier user interface and panel outputs (related to Figure 8)

a The MagicTPiT user interface allows selection of idat files via the "Browse…" button (long white arrow). After the upload is complete, clicking "Run" (short white arrow) initiates the analysis. Once processing has finished, the output panels from each analysis (black arrow) module can be accessed individually.

b-f Representative panel outputs are shown, including quality control metrics (b), beta-value densities (c), copy number profile (d), consensus heatmap (e), and calculated subgroup scores (f). The shown processed sample ("Sample_A") demonstrates good quality control metrics, abundant chromosomal alterations, and a match with subgroup 1.

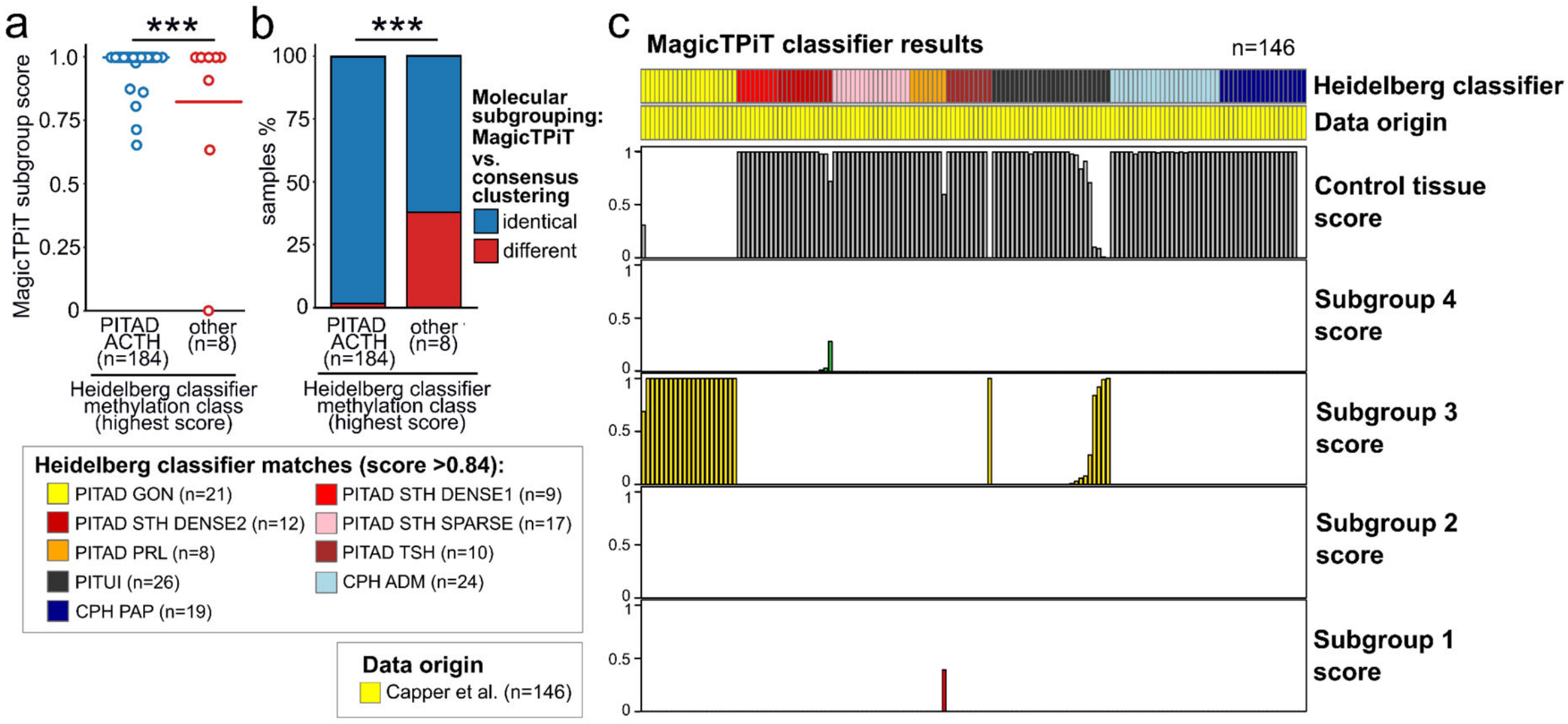

Supplementary Figure 9: Reliability and limitations of the MagicTPiT classifier (related to Figure 8)

a Tumour subgroup scores assigned by the MagicTPiT classifier were significantly lower in samples with highest Heidelberg classifier v12.8 [11] scores conforming to methylation classes other than PITAD ACTH. Two-sided Wilcoxon rank-sum. p=5.79e-06.

b Tumours with highest Heidelberg classifier v12.8 scores conforming to methylation classes other than PITAD ACTH more frequently showed different molecular subgroup assignments comparing highest MagicTPiT scores and original consensus clustering results. The results shown in a and b indicate that the combined use of the Heidelberg and the MagicTPiT classifier increases diagnostic power. Chi-square test. p= 2.026e-07.

c Previously published methylation data of sellar neoplasms [1] comprising non-corticotroph PitNETs and non-PitNETs were processed using the MagicTPiT classifier. Most of these sellar tumours matched best with anterior pituitary control tissue, except for gonadotroph PitNETs and a subset of posterior pituitary lobe tumours, which matched best with subgroup 3. These results highlight the limitations of the MagicTPiT classifier and showcase that this tool must only be used for histopathologically confirmed corticotroph PitNETs.

Heidelberg classifier class abbreviations: PITAD: pituitary adenoma; ACTH: adrenocorticotropic hormone; GON: gonadotropin; STH: somatotropin (growth hormone (GH)); DNS: dense; SPA: sparse; PRL: prolactin; TSH: thyroid stimulating hormone; PITUI: pituicytoma / granular cell tumour of the sellar region / spindle-cell oncocytoma; CPH: craniopharyngioma; ADM: adamantinomatous; PAP: papillary.

Sample sizes (n) indicate the number of tumour samples. Source data are provided as a Source Data file.

Supplementary References

1. Capper, D. *et al.* DNA methylation-based classification of central nervous system tumours. *Nature* **555**, 469–474 (2018).
2. Salomon, M. P. *et al.* The epigenomic landscape of pituitary adenomas reveals specific alterations and differentiates among acromegaly, Cushing's disease and endocrine-inactive subtypes. *Clin. Cancer Res.* **24**, 4126–4136 (2018).
3. Zhang, F. *et al.* Integrated proteogenomic characterization across major histological types of pituitary neuroendocrine tumors. *Cell Res.* **32**, 1047–1067 (2022).
4. Bujko, M. *et al.* USP8 mutations in corticotroph adenomas determine a distinct gene expression profile irrespective of functional tumour status. *Eur. J. Endocrinol.* **181**, (2019).
5. Neou, M. *et al.* Pangenomic Classification of Pituitary Neuroendocrine Tumors. *Cancer Cell* **37**, 123-134.e5 (2020).
6. Silva-Júnior, R. M. P. da *et al.* Integrating Methylome and Transcriptome Signatures Expands the Molecular Classification of the Pituitary Tumors. *J. Clin. Endocrinol. Metab.* **108**, 1452–1463 (2023).
7. Belakhoua, S. *et al.* DNA methylation profiling of pituitary neuroendocrine tumors identifies distinct clinical and pathological subtypes based on epigenetic differentiation. *Neuro. Oncol.* **27**, (2025).
8. WHO Classification of Tumours Editorial Board. Endocrine and neuroendocrine tumours. Lyon (France): International Agency for Research on Cancer; 2022. (WHO classification of tumours series, 5th ed.; vol. 8).
9. Trouillas, J. *et al.* A new prognostic clinicopathological classification of pituitary adenomas: A multicentric case-control study of 410 patients with 8 years post-operative follow-up. *Acta Neuropathol.* **126**, 123–135 (2013).
10. Jotanovic, J. *et al.* Genome-wide methylation profiling differentiates benign from aggressive and metastatic pituitary neuroendocrine tumors. *Acta Neuropathol.* **148**, 68- (2024).
11. Sill, M. *et al.* Advancing CNS tumor diagnostics with expanded DNA methylation-based classification. *Cancer Cell* **44**, 340-354.e2 (2026).
