# Supplementary material for: Multi-omics integration unravels four molecular subgroups of corticotroph pituitary neuroendocrine tumours with distinct clinicopathological features
Source: Nat Commun. 2026 Aug 4;17:7777. doi: 10.1038/s41467-026-76292-y (PMC13438112; doi:10.1038/s41467-026-76292-y)
Supplement: Supplementary file 4 [file 41467_2026_76292_MOESM2_ESM.pdf]

The Description of Additional Supplementary Files

Supplementary Data 1: Internal case series overview Supplementary Data 1 provides a sample-level overview of the clinicopathological annotations and molecular subgroup assignments for all internally derived cases (related to all Figures and Supplementary Figures).

Supplementary Data 2: External case series overview Supplementary Data 2 provides a sample-level overview of all externally derived cases and their molecular subgroups, as assigned in this study (related to Figures 5-8 and Supplementary Figures 3-9).

Supplementary Data 3a: Top 2500 variant cpgs across corticotroph PitNETs Supplementary Data 3a provides an overview of the top 2500 variant cpgs, which formed the basis for the analyses shown in Figure 1b and 1e.

Supplementary Data 3b: Top 250 variant genes across corticotroph PitNETs Supplementary Data 3b provides an overview of the top 250 variant genes, which formed the basis for the analyses shown in Figure 1c and 1f.

Supplementary Data 3c: Top 250 variant proteins across corticotroph PitNETs Supplementary Data 3c provides an overview of the top 250 variant proteins, which formed the basis for the analyses shown in Figure 1d and 1g.

Supplementary Data 4a: RNA-based gene set enrichment in subgroup 1 Supplementary Data 4a provides an overview of all significantly positively and negatively enriched gene sets in subgroup 1 compared to the other subgroups after p-values were adjusted for multiple comparisons using Bonferroni correction. The analysis was based on transcriptome data (related to Figure 2c).

Supplementary Data 4b: RNA-based gene set enrichment in subgroup 2 Supplementary Data 4b provides an overview of all significantly positively and negatively enriched gene sets in subgroup 2 compared to the other subgroups after p-values were adjusted for multiple comparisons using Bonferroni correction. The analysis was based on transcriptome data (related to Figure 2c).

Supplementary Data 4c: RNA-based gene set enrichment in subgroup 3 Supplementary Data 4c provides an overview of all significantly positively and negatively enriched gene sets in subgroup 3 compared to the other subgroups after p-values were adjusted for multiple comparisons using Bonferroni correction. The analysis was based on transcriptome data (related to Figure 2c)

Supplementary Data 4d: RNA-based gene set enrichment in subgroup 4 Supplementary Data 4d provides an overview of all significantly positively and negatively enriched gene sets in subgroup 4 compared to the other subgroups after p-values were adjusted for multiple comparisons using Bonferroni correction. The analysis was based on transcriptome data (related to Figure 2c).

Supplementary Data 5a: Stemness gene set used for single-sample gene set enrichment analyses Supplementary Data 5a provides a list of the 79 members of the “stemness” gene set (related to Figure 2d and Supplementary Figure 2b).

Supplementary Data 5b: Synapse assembly gene set used for single-sample gene set enrichment Supplementary Data 5b provides a list of the 48 members of the “synapse assembly” gene set (related to Figure 2d and Supplementary Figure 2c).

Supplementary Data 5c: Gonadotrophs gene set used for single-sample gene set enrichment analyses 1 Supplementary Data 5c provides a list of the 20 members of the “gonadotrophs” gene set (related to Figure 2d).

Supplementary Data 5d: Secretory vesicle gene set used for single-sample gene set enrichment analyses Supplementary Data 5d provides a list of the 16 members of the “secretory vesicle” gene set (related to Figure 2d and Supplementary Figure 2d)

Supplementary Data 6a: Protein-based gene set enrichment in subgroup 1 Supplementary Data 6a provides an overview of all significantly positively and negatively enriched gene sets in subgroup 1 compared to the other subgroups after p-values were adjusted for multiple comparisons using Bonferroni correction. The analysis was based on proteome data (related to Supplementary Figure 2a)

Supplementary Data 6b: Protein-based gene set enrichment in subgroup 2 Supplementary Data 6b provides an overview of all significantly positively and negatively enriched gene sets in subgroup 1 compared to the other subgroups after p-values were adjusted for multiple comparisons using Bonferroni correction. The analysis was based on proteome data (related to Supplementary Figure 2a)

Supplementary Data 6c: Protein-based gene set enrichment in subgroup 3 Supplementary Data 6c provides an overview of all significantly positively and negatively enriched gene sets in subgroup 1 compared to the other subgroups after p-values were adjusted for multiple comparisons using Bonferroni correction. The analysis was based on proteome data (related to Supplementary Figure 2a)

Supplementary Data 6d: Protein-based gene set enrichment in subgroup 4 Supplementary Data 6d provides an overview of all significantly positively and negatively enriched gene sets in subgroup 1 compared to the other subgroups after p-values were adjusted for multiple comparisons using Bonferroni correction. The analysis was based on proteome data (related to Supplementary Figure 2a)

Supplementary Data 7: Histopathological scoring results Supplementary Data 7 provides a sample-level overview of histopathological feature scorings for internal cases. The mean scorings as well as the individual scorings of two independent observers are shown (related to Figures 3-5 and Supplementary Figures 3 and 5).
